# An Evaluation Protocol for Subtype-Specific Breast Cancer Event Prediction

**DOI:** 10.1371/journal.pone.0021681

**Published:** 2011-07-08

**Authors:** Herman M. J. Sontrop, Wim F. J. Verhaegh, Marcel J. T. Reinders, Perry D. Moerland

**Affiliations:** 1 Molecular Diagnostics Department, Philips Research, Eindhoven, The Netherlands; 2 Delft Bioinformatics Lab, Delft University of Technology, Delft, The Netherlands; 3 Bioinformatics Laboratory, Department of Clinical Epidemiology, Biostatistics, and Bioinformatics, Academic Medical Center, Amsterdam, The Netherlands; 4 Netherlands Bioinformatics Centre, Nijmegen, The Netherlands; National Taiwan University Hosipital, Taiwan

## Abstract

In recent years increasing evidence appeared that breast cancer may not constitute a single disease at the molecular level, but comprises a heterogeneous set of subtypes. This suggests that instead of building a single monolithic predictor, better predictors might be constructed that solely target samples of a designated subtype, which are believed to represent more homogeneous sets of samples. An unavoidable drawback of developing subtype-specific predictors, however, is that a stratification by subtype drastically reduces the number of samples available for their construction. As numerous studies have indicated sample size to be an important factor in predictor construction, it is therefore questionable whether the potential benefit of subtyping can outweigh the drawback of a severe loss in sample size. Factors like unequal class distributions and differences in the number of samples per subtype, further complicate comparisons. We present a novel experimental protocol that facilitates a comprehensive comparison between subtype-specific predictors and predictors that do not take subtype information into account. Emphasis lies on careful control of sample size as well as class and subtype distributions. The methodology is applied to a large breast cancer compendium involving over 1500 arrays, using a state-of-the-art subtyping scheme. We show that the resulting subtype-specific predictors outperform those that do not take subtype information into account, especially when taking sample size considerations into account.

## Introduction

Breast cancer *event prediction* is an important yet challenging classification problem in which one attempts to predict whether a certain type of event will happen within a given time frame or not, e.g. whether a breast tumor will metastasize or not, based on gene expression data obtained from microarrays. A well-known example of such a predictor is the 70-gene signature by van't Veer et al. [1]. In recent years increasing evidence appeared implying that breast cancer may not constitute a single disease at the molecular level, but that breast cancers comprise a diverse and heterogeneous set of diseases [2].

Various breast cancer subtyping schemes have been proposed, mostly inspired by the *intrinsic gene list* approach from the landmark publication by Perou et al. [3]. The latter introduced a breast cancer subtype taxonomy that classifies breast cancers as either luminal A (lumA), luminal B (lumB), basal, Her2 or normal-like, based on hierarchical clustering. A more recent example is a subtyping scheme based on a biology-inspired module-driven approach [4], that identifies the subtypes lumA, lumB, basal, and Her2 through model-based clustering. The precise definition of the subtypes themselves and of a standardized geneset to classify samples to a specific subtype is still subject of debate. Several studies indicated stability issues with the intrinsic gene list approach [5]–[7]. Furthermore, doubts have been casted on the existence of the normal-like tumors as a genuine breast cancer subtype [8]. Despite this debate, it is widely accepted that over large sample sets breast cancer subtypes are associated with a difference in survival time. This suggests that instead of using a single monolithic predictor, better prognostic predictors might be constructed that solely target samples of a designated subtype. However, only few studies couple subtyping directly to breast cancer event prediction [8]–[10]. In this paper we address the question whether predictors targeting a specific subtype, referred to as *typed* predictors, can outperform *untyped* predictors that do not take subtype into account. The main contribution of this work is the definition of a novel experimental protocol which explicitly addresses three main problems of such a comparison, i.e. subtype definition, sample size, and class imbalance.

### Subtype definition

In this paper we are interested in the possibilities of improving microarray breast cancer event prediction by exploiting subtype information. A core ingredient of our protocol is the construction of a sequence of subtype-specific predictors that via systematic pooling steps gradually transform into an untyped baseline predictor.

**Figure 1:**
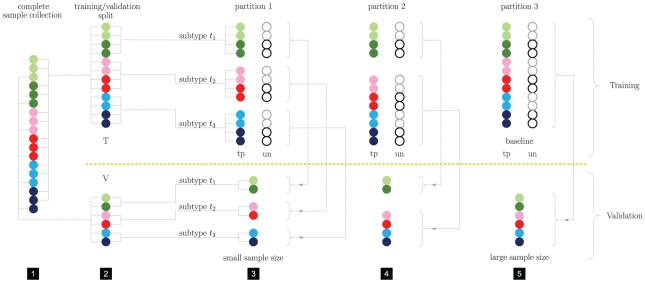
Conceptual overview of the stratification protocol. 1) toy sample set, comprised of three subtypes (blue, red and green), lighter (darker) shades indicate positive (negative) cases. 2) stratified split (by class label and subtype) of the data into a training set 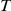 and a validation set 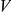. For each set separately various partitions are created. The yellow dashed line illustrates the strict separation of training (top) and validation (bottom) parts. 3) the most refined partition involves a single subtype per part. The typed version (tp) partitions 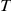 by parts stratified by class label and subtype. The untyped (un) counterpart involves parts stratified by class label only, however, each untyped part involves an identical number of positive and negative training samples as its typed counterpart. Here lighter (darker) open circles represent positive (negative) cases. Alternative partitions can be constructed by pooling some or all of the initial parts, as depicted in 4) and 5). On each training part a separate predictor is constructed, which is evaluated on a specific set of validation samples. Note that paired typed and untyped predictors are evaluated on the same set of validation samples. 5) presents a special case for which typed and untyped training sets are identical and equal the overall training set 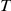. This set is used to construct the baseline predictor. The untyped predictors associated with partitions 1 and 2 represent down-scaled versions of the baseline and serve to assess the influence of sample size.

A conceptual overview of the stratification of subtypes is provided by Figure 1. From the application of a given subtyping scheme, e.g. the module-based approach of Desmedt et al. [4], each sample is associated with a specific subtype. These subtype labels are subsequently used to construct various partitions of the available data. For each part of a partition a separate predictor is constructed, which targets a specific subset of samples. The most refined partition contains one subtype per part. From this partition a sequence of alternative partitions is created by systematic pooling of individual parts. Ultimately, this leads to a partition with a single part. The performance of this partition serves as a natural baseline as its associated predictor is essentially untyped and is constructed on the largest sample set available, which simultaneously represents the most heterogenous set w.r.t. to the selected subtyping scheme. For a given partition, of interest are the performance per part, as well as the overall performance associated with it, that is, the performance as evaluated over all available samples. We note that, even though the set of subtypes used to construct partitions is of great interest, its precise makeup is of a lesser concern in this paper, as we are mainly concerned in setting up a proper comparison between partitions.

### Sample size

The sample size problem manifests itself in different ways. Firstly, stratification by subtype drastically reduces the size of the sample set available for the construction of typed predictors (Figure 1). As numerous studies have shown that a larger sample size leads to better performance [11]–[13] it is therefore non-trivial if the potential benefit of subtyping can outweigh a severe loss in sample size. Secondly, differences in sample size per subtype also complicate the comparison between typed predictors. This imbalance is clearly illustrated by the application of a state of the art model-based subtyping scheme [4] to a compendium of 892 breast cancer samples (Table 1) used in this paper. Our experimental protocol strongly controls these sample size effects to enable a systematic comparison of typed and untyped predictors.

**Table 1:** Compendium subtype distribution.

	lumA	lumB	basal	Her2	*D*
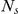	273 (41.2)	216 (32.8)	100 (15.1)	74 (11.2)	663 (100)
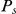	42 (18.3)	94 (41.0)	57 (24.9)	36 (15.7)	229 (100)
total	315 (35.3)	310 (34.8)	157 (17.6)	110 (12.3)	892 (100)
ratio	6.5	2.8	1.8	2.1	2.9

Distribution of class labels and subtypes for the 892 samples with a proper class label. 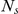 and 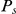 denote the number of negative (good prognosis) and positive (poor prognosis) cases of for each subtype 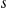, *total* and *ratio* represent the sum and ratio of 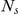 and 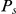, respectively. Entries in brackets indicate percentages w.r.t. the entire compendium (column *D*).

### Class imbalance

Imbalance with respect to the class label distribution is another important characteristic of many cancer related datasets. Also in our breast cancer compendium the positive class, i.e. the poor prognosis group, is much smaller than the negative class, i.e. the good prognosis group (Table 1, column *D*). Such imbalance often negatively affects the performance of a predictor for the minority class. The literature offers several solutions for the class imbalance problem. Popular approaches are to either undersample the majority class, to oversample the minority class, or to adapt the cost structure [14], [15]. This is especially important in a subtyping setting where a proper comparison of predictors is affected by a class imbalance inherent to the subtyping itself. Note that, if the subtype has a profound impact on the survival rate, we expect distinct subtypes to be associated with different negative to positive class ratios. In our compendium, we see that this is indeed the case (Table 1). Comparisons between predictor performances using frequently adopted performance measures like accuracy, positive and negative predicted value, can easily be obscured by a difference in the class ratio. For these reasons, proper balancing is essential.

In this paper, we present an experimental evaluation protocol that highly facilitates the comparison between typed and untyped predictors, in which sample size as well as class and subtype distributions are controlled and by which their individual contributions can be properly studied. In order to facilitate a proper comparison, besides working with the complete (unbalanced) compendium, we also consider performance on a set of *balanced* compendia which have the same sample size and negative-positive class ratio for each subtype and are obtained via undersampling of the majority class. Although here applied to microarray breast cancer event prediction, the methodology is also applicable to other types of diseases or data obtained by alternative measurement techniques.

## Materials and Methods

In the following we present a predictor construction and evaluation protocol to investigate the potential of typed prediction and its relation to sample size. The protocol produces a sequence of predictors that via systematic pooling steps gradually transform into an untyped baseline predictor. As appropriate choices for a prediction rule, ranking, subtyping strategy, and performance measure are domain-specific, for the moment we assume they are given.

### Partitioning scheme

**Figure 2:**
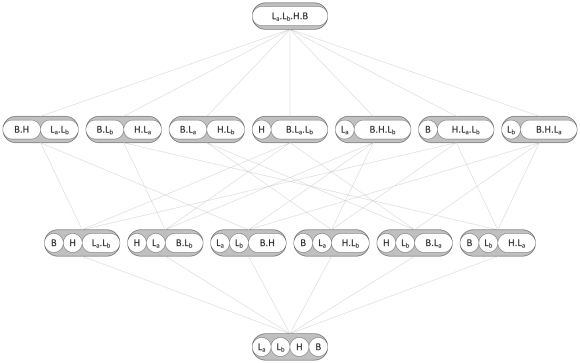
Partitioning scheme. The Hasse diagram depicts all possible partitions (grey ovals) w.r.t. an example breast cancer subtype set 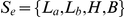, representing the subtypes lumA, lumB, Her2, and basal, respectively. White ovals indicate parts. The lines represent a move from one partition to another by either merging two parts (bottom to top) or splitting one part into two parts (top to bottom). The top layer depicts the coarsest partition in which all elementary types have been pooled into a single part, making it essentially untyped. The bottom layer represents the most refined partition, i.e. one part for each elementary subtype. For each distinct part a separate predictor is constructed. The partition in the top layer is used for baseline predictor construction. In this example 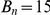, 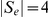, 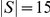 and 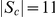.

Let 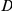 denote the set of all available samples with proper event data, that are associated with a set of 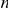 *elementary* subtypes 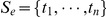. The elementary subtypes form the most obvious candidates to consider for typed prediction. In this case one would partition the available sample set 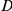 into exactly 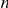 parts. Less refined partitions, however, can be considered by pooling members of several elementary subtypes, ultimately leading to a single part, that is essentially untyped. Let 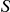 denote the collection of distinct parts over all partitions, that is, the powerset of 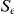 minus the empty set with cardinality 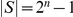. We will refer to the set 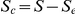 as the set of *compound* subtypes, the members of which are comprised of several of the elementary subtypes. In general, the number of distinct partitions is given by the 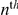 Bell number [16], denoted by 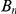, where 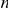 represents the number of elementary subtypes. The complete set of partitions can be conveniently arranged into a Hasse diagram, see Figure 2, which shows an example for 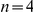 elementary subtypes.

### Evaluation protocol and predictor construction

In essence our evaluation protocol can be seen as an extension of the protocol proposed by Wessels et al. [17]. Our protocol consists of a repeated stratified cross-validation scheme for the typed predictors, after which we deliberately randomize the corresponding training sets w.r.t. subtype distribution, in order to obtain results for the untyped predictors. Below we give a formal description of the protocol.

#### Notation

**Figure 3:**
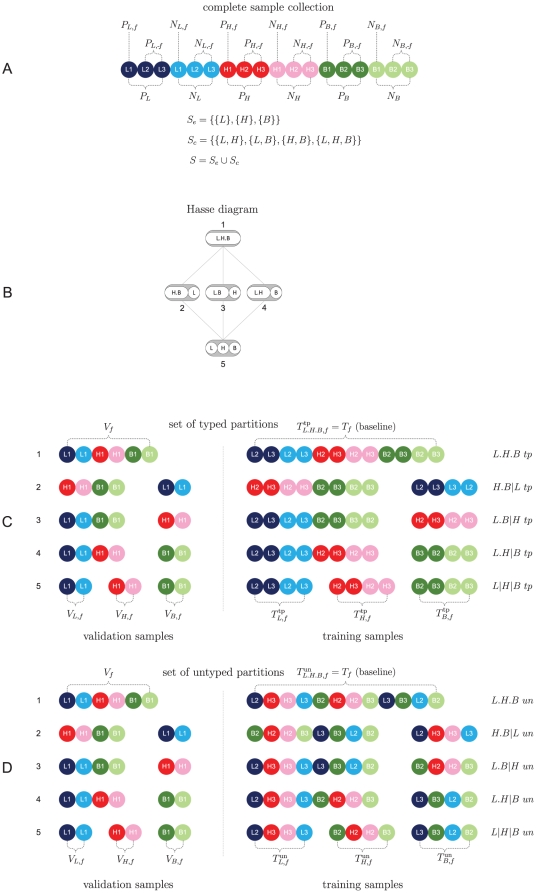
Stratification toy example. For a detailed explanation, see the running text.

Let 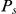 and 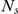 denote the sets of positive and negative samples of subtype 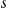. For each 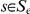 we divide the corresponding sets 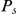 and 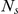 into 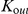 folds of approximately the same size. Let 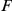 denote the set of all folds, with 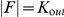, let 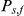 (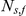) denote fold 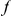 of 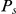 (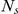) and let 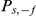 (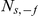) denote the union of all folds but fold 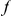. Now we can define the training and validation sets for typed and untyped predictors. A detailed toy example clarifying the sets as defined in the following two subsections is provided by Figure 3.

#### Typed sets

For each elementary subtype 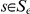 and fold 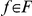 we construct a typed training set 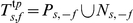 and a validation set 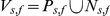. Furthermore, for each compound subtype and fold we pool the training and validation sets of the subtypes that comprise it, that is, for compound subtype 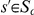 consisting of the elementary subtypes 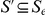 we have 
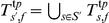
 and 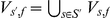.

#### Untyped sets

In order to construct untyped counterparts of the typed training sets let 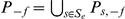 and 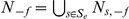. For each elementary subtype 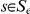 and fold 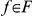 we create the sets 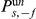 and 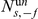 by randomly drawing *without replacement* 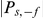 positive and 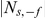 negative samples from the sets 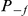 and 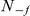, respectively. Analogously to the typed scenario, for each elementary subtype 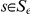 and fold 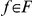 we next construct an untyped training set 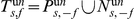, which has the same negative to positive ratio as 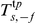. Finally, for each compound subtype and fold we again pool the corresponding training sets of the elementary subtypes that comprise it, that is, for compound subtype 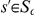 consisting of the elementary subtypes 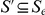 we have 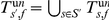. Typed and untyped predictors are paired and their performance is evaluated on the same validation set.

#### Baseline

Note that the only partition for which typed and untyped sets are identical is the partition in which all elementary subtypes have been pooled into one part. In this case typed and untyped predictors for each fold 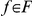 are associated with the same training set 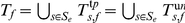, with corresponding validation set 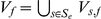. We will refer to these predictors as *baseline predictors*.

#### Toy example visualizing the construction of typed and untyped set

Consider the balanced toy dataset depicted in Panel A) of Figure 3, which is an extension of the example depicted in Figure 1. The sample set is again comprised of three three elementary subtypes, 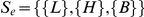, representing for instance the subtypes luminal (blue), Her2 (red), and basal (green), respectively. Each elementary subtype consists of three positive (poor prognosis) cases, depicted by darker shades and three negative (good prognosis) cases, depicted by lighter shades. Instead of an individual sample (Figure 1), here each circle corresponds to multiple samples. Panel B) depicts the associated Hasse diagram w.r.t. the elementary subtype set 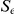 with five partitions (see also Figure 2). Panel C) presents an overview of the five typed partitions of the Hasse diagram in the context of a 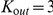-fold cross-validation scheme. The example depicts the sets associated with a single fold. Validation sets are depicted at the left of the vertical dotted line, training sets on the right. Each part in a partition is depicted as a connected string of filled circles. For each training part a separate predictor is constructed. Partition names are given at the outer right, where a dot indicates pooling, and a vertical dash is used to separate parts. Finally, Panel D) depicts five untyped partitions for a single fold. The untyped training set for the most refined partition (#5) is constructed from the typed training set by randomly swapping light shaded training instances with each other and dark shaded instances with each other. This guarantees that the negative-positive class ratio is the same for typed and untyped sets. Coarser partitions (#1–4) are formed by combining parts according to the Hasse diagram of panel B. Note that for the coarsest partition (#1), typed and untyped training sets are identical. This set is used for the construction of the baseline predictor. Last, note that typed and untyped partitions are always associated with the same set of validation samples. Furthermore, training and validation samples are always strictly separated.

#### Training protocol

On every training set we invoke an identical training protocol, which is a mild adaptation of the protocol proposed by Wessels et al. [17]. Let 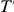 denote the set of available training samples. In a first step we divide 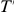 into 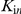 folds stratified w.r.t. class label and subtype. For each fold 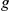 we perform a ranking using the learning set 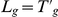, after which we construct a sequence of 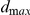 predictors 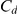 using the top 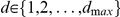 ranked features on 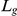. We then employ these predictors to predict the events corresponding to the evaluation set 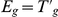 and subsequently aggregate the results over all folds from which we construct a performance curve, which for a performance indicator of interest tells us the performance for a given number of features, up to 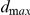. The previous training steps are repeated 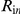 times in order to construct an average performance curve which for a given set size reports the average performance over all repeats. We refer to this loop as the *inner loop* of our protocol.

Let 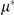 denote the maximum value of the average performance curve and denote its standard deviation over 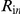 repeats by 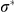. Since larger signatures are often more robust [18], we take the optimal number of features to be the largest integer 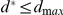 such that its associated training performance 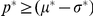. Finally, we use the full training set 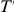 to rank the available features and construct a predictor 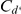 using the top 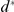 ranked features on 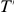 and conclude by returning 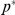, 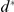, as well as the trained predictor 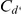. In addition to an optimized signature size 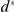, a fixed size can be considered as well.

#### Performance evaluation

For each subtype 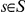 and for each fold 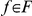 we invoke the training protocol on the typed and untyped training sets, 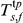 and 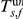, and apply both of the resulting predictors to the same validation set 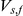. Let 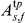 and 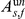 denote the assignments made on this validation set by the typed and untyped predictors, respectively. For each subtype 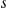 we construct a subtype-specific performance indicator for the typed and untyped predictors by considering the aggregated assignments over all folds 
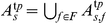
 and 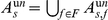. Finally, for a given partition 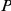 we obtain an overall performance estimate for typed and untyped predictors by considering the aggregated assignments over all its parts 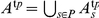 and 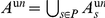, respectively. To compensate for sampling effects all previous steps are repeated 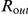 times, after which we average performance indicators over all repeats. We refer to this loop as the *outer loop*.

#### Schematic representation main evaluation protocol

**Figure 4:**
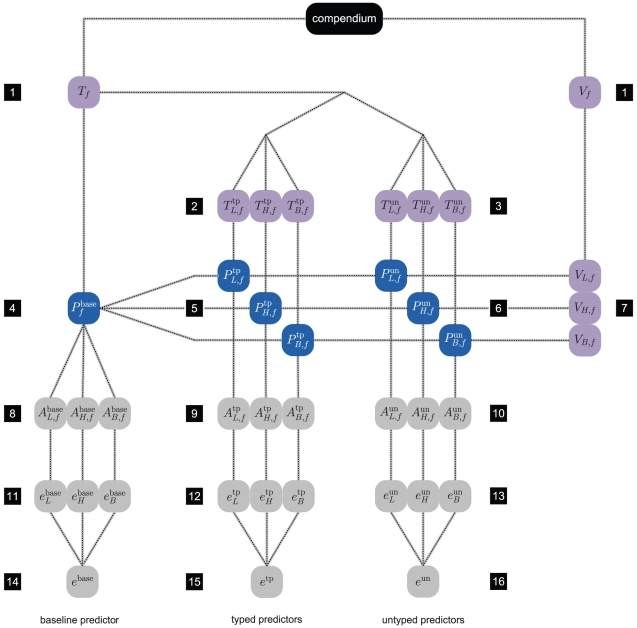
Bird's eye view of evaluation protocol. For additional details, see running text. 1) Stratified split w.r.t. class label and subtype of the complete data set in a training set 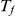 and a validation set 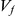. 2) Construction of typed training sets 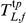, 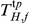 and 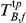. 3) Construction of untyped training sets 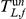, 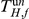 and 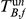. 4) Baseline predictor construction. 5) Typed predictor construction. 6) Untyped predictor construction. 7) Stratification of validation set by subtype. 8) Invoke baseline predictor on validation samples. 9) Invoke typed predictors on associated validation samples. 10) Invoke matching untyped predictors on same validation sets. Steps 1–10 are repeated for all folds 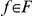. 11–13) Subtype-specific performance estimation based on the aggregated event predictions (over all folds) per subtype, as made by the baseline (11), typed (12), and untyped (13) predictors. 14–16) Overall performance estimation based on the aggregated event predictions over all folds made by the baseline (14), typed (15), and untyped (16) predictors.

Figure 4 presents a schematic representation of the main evaluation protocol as described above when applied to the toy dataset example of Figure 3. For clarity the figure depicts the scenario for a single fold 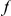 and depicts only two of the 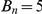 partitions i.e. the coarsest (partition 1, Figure 3) and the most refined (partition 5, Figure 3). The former partition is associated with the baseline predictor, for which typed and untyped are identical and involves steps 1, 4, 8, 11, and 14 of Figure 4. The second partition contains one part for each elementary subtype. Typed predictors involve steps 2, 5, 9, 12, and 15, while untyped predictors involve steps 3, 6, 10, 13, and 16.

### Performance measures

Class imbalance influences the choice of a suitable performance measure. Comparison of performance by the total accuracy rate has the disadvantage that a predictor that always guesses the majority class is associated with a high performance, while in fact it misclassifies the complete minority class. A more appropriate performance measure is the area under the ROC curve, which is insensitive to varying class proportions. Also the *balanced accuracy rate*, defined as the average of the sensitivity and specificity of the prediction rule, has been used in an imbalanced setting [12], [17], [19]. This measure has the advantage that we can no longer achieve a high performance by sacrificing one class for another, as doing so results in a performance equal to that obtained by random guessing, i.e. a balanced accuracy rate of 50%.

Our main performance indicator is the area under the ROC curve (*auc*). We also report the balanced accuracy rate (*bar*) and the accuracy (*acc*). Since summarizing predictor performance on both classes in a single measure causes loss of information, we also report four other frequently used performance indicators that report performance for a proper subset of the samples: sensitivity (*sen*), specificity (*spc*), positive predictive value (*ppv*), and negative predictive value (*npv*). For a thorough overview of these and other performance indictors see [20].

### Balanced compendia

Since the number of samples and the negative-positive class ratio differ considerably per subtype (Table 1), we constructed a set of balanced compendia that are properly stratified w.r.t. the class ratio. Note that the largest sample set that can be constructed with the same number of samples and the same ratio 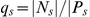 for all elementary subtypes can hold at most 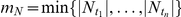 negative samples and 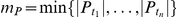 positive samples. Therefore, in order to obtain a balanced compendium 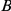, we randomly draw *without replacement* 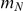 negative samples from 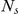 and 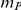 positive samples from 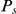 for each elementary subtype 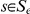. Let 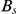 denote the set of 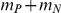 samples drawn for subtype 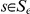, then 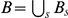. Since for most elementary subtypes the sampling can be done in multiple ways, we repeat the sub-sampling process 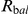 times. Note that, compared to the unbalanced compendium 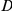, the balanced compendia 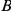 are well controlled w.r.t. subtype distribution, sample size, and class distribution.

### Compendium construction

The compendium pools data of ten individual microarray datasets. All datasets were measured on the same platform (Affymetrix HG-U133A). This circumvents the need for cross-platform normalization, which can be challenging [21]. All raw expression data used is publicly available in the MIAME compliant databases Gene Expression Omnibus (GEO) [22] and ArrayExpress [23] and can be found under the following accession numbers: GSE2034 [10], GSE5327 [24], GSE7390 [25], GSE11121 [26], GSE2603 [27], GSE6532 [28], GSE2990 [29], GSE3494 [30], GSE1456 [31], and E-TABM-158 [32]. All accession numbers represent GEO accession numbers, with exception of E-TABM-158 [32], the expression data of which is stored at ArrayExpress. After removing duplicate entries and outlier arrays, detected using the *arrayQualityMetrics* package [33], 1539 unique hybridizations remained. Raw expression data was used to generate MAS5.0 expression estimates, using the *affy* package, scaled to a target intensity of 600. Prior to pooling expression data, the expression estimates were 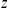-transformed for each study and each gene separately, as suggested in [21], [34]. For event prediction purposes, all class labels are solely based on a single type of survival data, being distant metastasis free survival (dmfs). *Poor prognosis* cases (PP) had an event, i.e. distant metastasis within five years, while the *good prognosis* cases (GP) did not have an event during follow-up, with a follow-up time of at least five years i.e. samples with an event after five years were removed. These stringent criteria led to the identification of 229 PP samples and 663 GP samples, yielding a total of 892 unique samples. A list of the individual CEL file identifiers is presented in Supporting Information S1.

### Subtyping scheme

Subtyping is based on a recently introduced biology-inspired module-driven approach [4], that identifies the subtypes lumA, lumB, basal, and Her2 through model-based clustering. In contrast to the intrinsic gene list approach [3], clustering is not performed on the expression data directly. Instead the expression values are first projected onto a lower dimensional space, in which each sample is represented by three *module scores* related to key biological processes strongly associated with breast cancer. The modules consist of an ER-related module, comprising 469 genes, a Her2-related module of 28 genes, and a proliferation-related module, referred to as AURKA, containing 229 genes. After transformation of the expression data to module scores, a Gaussian mixture model is fitted on the module data in order to determine the cluster membership of each sample. ER and Her2 module scores are used to infer the subtypes luminal, Her2, and basal, while the AURKA module is used to further subdivide the luminal group into a lumA and a lumB group.

In order to obtain the most likely subtype assignment for each sample, we estimated the subtype model on the set of all 1539 available samples. This resulted in 564 (36.8%), 543 (35.4%), 246 (17.6%) and 186 (16.1%) assignments to the subtype categories lumA, lumB, basal, and Her2, respectively. Table 1 presents an overview of these assignments for the set of 892 samples with properly defined class labels. The subtype distribution over the 892 sample set is similar to the subtype distribution over the complete compendium with 35.3%, 34.8%, 17.6%, and 12.3% belonging to the subtypes lumA, lumB, basal, and Her2, respectively (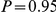, Pearson's chi-square test). Subtyping was performed using the *genefu* package.

### Balanced sets

From Table 1 it follows that in order to obtain a fully balanced compendium, we can select at most 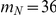 negative and 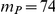 positive cases for each 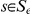, which in turn implies 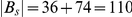 and 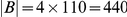.

### Protocol implementation details

In this paper results are reported over a set of 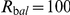 balanced breast cancer compendia, and for an unbalanced compendium of 892 samples. For the inner loop we employed 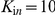-fold cross-validation, with 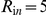 repetitions. Predictors are based on the nearest centroid (NC) rule, which despite its simplicity often shows good performance. Furthermore, a NC is known to be reasonably noise tolerant [17]. As a distance measure the cosine correlation distance was used. For each separate fold of the training set we first performed a filtering step, using the present/absent calls from the MAS5.0 procedure and only selected genes for which in at least one of the positive or negative sample groups the number of present calls was at least 70% [35]. The remaining features were ranked based on moderated-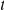 statistics, as implemented in the *limma* package [36], [37]. For predictor construction we considered average performance curves up to 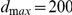 features, similar to van Vliet et al. [12]. Finally, in the outer loop we employed 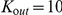-fold cross-validation, with 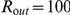 repetitions. ROC curves were generated by using the difference between the distance of a sample to each of the centroids as a continuous criterion, on which a variable threshold was set.

### Computing environment

In order to perform a comprehensive analysis many re-samplings of the data were performed, under various conditions. As for each re-sampling and for each part in the set of generated partitions separate predictors were constructed and evaluated, the complete analysis was computationally demanding. The methodology, however, lends itself well to parallelization. In order to perform our computations we used a grid involving 1648 cores, divided over 206 Dell PowerEdge blade servers, each with 2 Intel XEON L5420 Quadcore CPU's, with 16GiB FDB Dual Rank memory. All computations were performed using R [38] and Bioconductor [39].

## Results

### Improved auc and bar by typed prediction

**Figure 5:**
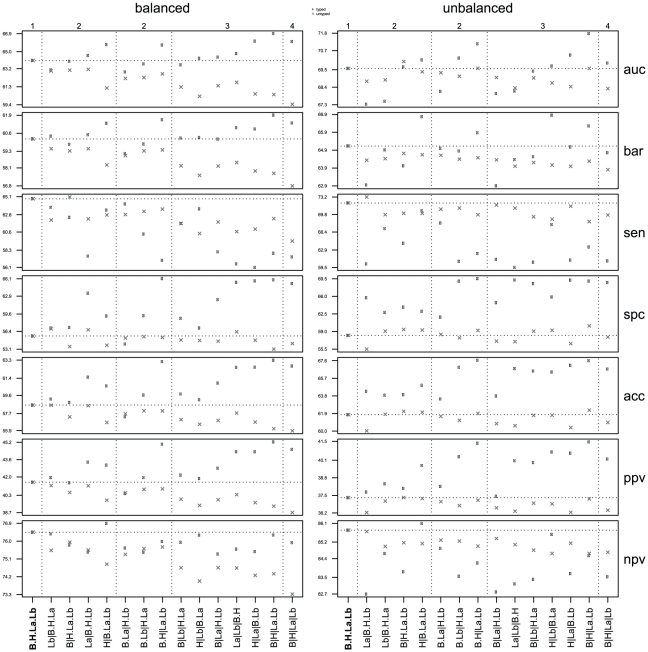
Overall performance overview for all partitions. Performance overview of overall performance corresponding to the 15 distinct partitions w.r.t. the elementary subtype set 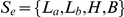, that represents the subtypes lumA, lumB, Her2 and basal, respectively (Figure 2). The left panel corresponds to experiments involving the balanced compendia 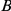, while the right panel corresponds to experiments involving the full unbalanced compendium 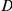. In each panel the top numbers 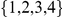 indicate the number of different parts in each of the partitions, while the bottom line identifies the precise makeup of the various partitions e.g. the notation B
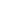
H
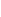
La.Lb indicates a partition into three parts, involving separate basal and Her2 groups, while having a combined luminal group. In each panel the coarsest partition is situated at the outer left, which corresponds to the baseline predictor (indicated in bold), that is, a single predictor that targets all samples. The most refined partition is situated at the outer right, which uses a separate predictor for each elementary subtype. A horizontal dotted line indicates the performance of the baseline predictors. Vertical dotted lines are used to group the partitions by their number of parts, as indicated by the top numbers. Results represent averages over 100 repeats. Rows represent seven frequently used performance indicators: area under curve (*auc*), balanced accuracy (*bar*), sensitivity (*sen*), specificity (*spc*), accuracy (*acc*), positive predictive value (*ppv*) and negative predictive value (*npv*). Performance for typed predictors is indicated with a dot, performance for untyped predictors with a cross.

Figure 5 depicts a condensed overview of overall performance corresponding to typed and untyped event predictors under various partitioning schemes, involving signatures based on the nearest centroid rule. Similar results were obtained using a signal-to-noise ratio ranking strategy, using 3-fold, 5-fold, and leave-one-out cross-validation instead of 10-fold cross-validation, or when using a more complex non-linear predictor (random forest [40]), see Supporting Information S2. A complete overview of the performance per subtype associated with Figure 5 is given in Supporting Information S3.

#### Performance on balanced compendia

The left panel in Figure 5 shows that typed predictors generally obtain a higher overall performance than their untyped counterparts on balanced compendia. The typed *auc* and *bar* are consistently higher, sometimes quite substantially. Furthermore, we see that *auc* and *bar* are well correlated.

One of the more interesting partitions is the one that uses a single part for each elementary subtype, which is situated at the outer right in each panel and corresponds to the partition depicted at the bottom of the Hasse diagram (Figure 2). In this partition overall performance in the typed case is obtained by employing four distinct typed predictors, each targeting a different part of the partition. Similarly, untyped overall performance is achieved by employing four downsized versions of the baseline predictor, in which each predictor is constructed on an equal number of good and poor prognosis samples as their typed counterparts. This is indeed one of the best performing partitions, with an associated overall *auc* and *bar* of 66.1% and 61.3% for the typed predictors, respectively, compared to 59.4% and 56.8% for the untyped predictors.

A more detailed overview corresponding to this partitioning with a breakdown of performance per subtype is given in Table 2. The subtype distribution of the training data indeed has a considerable impact on the performance of a predictor. Especially the Her2 group benefits from using a typed prediction rule with an *auc* and *bar* of 74.7% and 71.5%, respectively, for the typed predictors, compared to 65.9% and 61.7% for the untyped predictors. Results show an improvement for almost all other performance indicators as well when using typed predictors over untyped predictors, although for some subtypes untyped predictors achieve a higher sensitivity.

**Table 2:** Subtype-specific performance overview (balanced compendia).

		lumA	lumB	basal	Her2	overall
	auc	***61.5***	***65.0***	***60.6***	***74.7***	***66.1***
	bar	***56.3***	***60.8***	***56.7***	***71.5***	***61.3***
	sen	37.5	***71.7***	44.6	***75.9***	57.4
tp	spc	***75.1***	*49.8*	***68.8***	***67.2***	***65.2***
	acc	***62.8***	***57.0***	***60.9***	***70.0***	***62.7***
	ppv	***42.4***	***41.2***	***40.9***	***52.9***	***44.5***
	npv	*71.2*	***78.4***	*72.0*	***85.1***	***75.9***
	auc	55.3	60.6	57.1	65.9	59.4
	bar	53.8	57.0	54.7	61.7	56.8
	sen	***56.3***	66.4	*48.1*	67.0	***59.5***
up	spc	51.3	47.5	61.3	56.5	54.1
	acc	52.9	53.7	57.0	59.9	55.9
	ppv	36.1	38.1	37.9	43.1	38.7
	npv	70.7	74.7	70.9	77.9	73.3

Performance overview per elementary subtype: typed (tp) versus untyped (un) predictors on balanced compendia 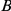. The highest value for a paired typed and untyped performance measure is set in italic. If the difference is significant (two sided paired 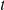-test, 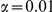) the entry is set in bold. Values in the column *overall* correspond to the overall performance depicted in the left panel of Figure 5.

The best overall performance is obtained by typed prediction using a partition which has separate Her2 and basal groups, and a combined luminal group (Figure 5, left panel, second partition from the right). This partition gives an overall *auc* and *bar* of 66.9% and 61.9%, respectively, compared to 60.5% and 57.7% for the untyped predictors.

Note that coarser partitions involve predictors for compound subtypes that are constructed on larger sample sets compared to those in more refined partitions. Increase in sample size can indeed be beneficial, as the baseline predictor, which is constructed on the largest training set possible under the given cross-validation scheme, is associated with the highest overall performance over all untyped predictors with an *auc* and *bar* of 64.1% and 60.2%, respectively (Figure 5). However, its performance is still lower than that obtained by using more refined typed prediction schemes. This clearly illustrates that a predictor trained on more samples without control for subtype distribution is not necessarily the optimal choice.

Finally, the increase in overall performance of typed predictors, as measured by *auc* and *bar*, is often accompanied by trading sensitivity for specificity. Compared to untyped predictors, typed predictors are generally associated with much higher specificity, yet lower sensitivity. Note that the highest sensitivity is in fact obtained by the baseline predictor.

#### Performance on unbalanced compendium

The right panel of Figure 5 reveals a similar pattern for typed and untyped prediction on an unbalanced compendium as seen in the left panel. Note that in contrast to the balanced sets 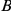, the set 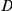 is unbalanced w.r.t. subtype distribution and is dominated by luminal samples (Table 1), hence performance on these samples drives overall performance. As expected, since most parts in the various partitions now contain a considerably larger number of samples compared to the balanced scenario, overall performance in terms of *auc* and *bar* improves. Similar to the balanced case, the highest overall performance is obtained by using a partition which has separate Her2 and basal groups, while using a combined luminal group. This partition has an *auc* and *bar* of 71.8% and 66.3%, respectively, which again outperforms the baseline predictor, which has an associated *auc* of 69.6 and 65.1%.

Table 3 is the unbalanced counterpart of Table 2. For the typed predictors an increase in sample size is indeed beneficial, as the *auc* and *bar* for all subtypes but Her2 increase. Note that the Her2 group in both the balanced and unbalanced case has the same size, hence its performance in the typed case remains unchanged. Furthermore, the most refined typed prediction scheme again outperforms its untyped counterpart, with an overall *auc* and *bar* of 69.9% and 64.8%, compared to 68.3% and 63.8%.

**Table 3:** Subtype-specific performance overview (unbalanced compendium).

		lumA	lumB	basal	Her2	overall
	auc	***64.8***	***71.9***	***62.2***	***74.7***	***69.9***
	bar	***56.3***	***64.7***	***58.0***	***71.5***	***64.8***
	sen	***31.3***	74.6	50.0	*75.9*	60.8
tp	spc	81.3	***54.7***	***66.1***	***67.2***	***68.8***
	acc	74.6	***60.7***	***60.2***	***70.0***	***66.7***
	ppv	20.5	***41.8***	***45.6***	***52.9***	***40.2***
	npv	***88.5***	83.2	***69.9***	***85.1***	83.5
	auc	63.0	70.2	50.4	60.3	68.3
	bar	54.6	62.3	50.9	57.5	63.8
	sen	19.9	***82.7***	***81.7***	74.9	***69.7***
up	spc	***89.2***	41.9	20.1	40.2	57.9
	acc	***80.0***	54.3	42.4	51.5	60.9
	ppv	***22.4***	38.3	36.8	37.9	36.4
	npv	87.9	***84.8***	65.6	76.7	***84.7***

Performance overview per elementary subtype: typed (tp) versus untyped (un) predictors on the unbalanced compendium 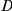. The highest value for a paired typed and untyped performance measure is set in italic. If the difference is significant (two sided paired 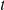-test, 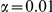) the entry is set in bold. Values in the column *overall* correspond to the overall performance depicted in the right panel of Figure 5.

For the untyped predictors, however, the story is more complex. Table 3 shows a substantial gain in overall performance for the untyped predictors, compared to the untyped overall performance of Table 2, with an *auc* and *bar* of 68.3% and 63.8%, respectively, compared to 59.4% and 56.8% on the balanced compendia. Although we see a substantial improvement in *auc* for lumA and lumB, for basal and Her2 we observe a considerable deterioration. However, since luminal samples dominate the subtype distribution in the unbalanced case, overall performance for untyped prediction still improves quite strongly compared to the balanced scenario. In addition, a striking difference between the sensitivity and specificity of the lumA subtype compared to the other subtypes can be observed.

### A dissection of the baseline performance

Table 4 presents a more detailed overview of how the baseline predictor obtains its performance. The baseline predictor shows an even more extreme difference between sensitivity and specificity, with a very high specificity for the lumA subtype of 97.9%, yet with a very low sensitivity of 5.8%. However, the sensitivity over the remaining subtypes is very high with values of 87.8%, 86.8% and 84.9% for the subtypes lumB, basal, and Her2, respectively. Apparently, the unbalanced untyped predictors are biased to predict a good prognosis for lumA samples, yielding a very high specificity but very poor sensitivity for that subtype, and to predict a poor prognosis for the other subtypes, yielding a high sensitivity but a rather low specificity for them. Finally, we note the peculiar behavior of the *bar* performance indicator in an unbalanced setting. The overall *bar* is 65.1%, however, for *every* individual subtype the corresponding *bar* is less, even though they form a partition of the complete sample set 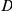. The same phenomenon can be seen for the untyped predictors of Table 3.

**Table 4:** Baseline predictor performance.

	lumA	lumB	basal	Her2	overall
auc	***68.6***	***72.7***	50.4	60.6	69.6
bar	51.8	63.2	49.5	58.1	***65.1***
sen	5.8	***87.8***	***86.8***	***84.9***	***72.0***
spc	***97.9***	38.6	12.2	31.3	58.2
acc	***85.6***	53.5	39.3	48.8	61.8
ppv	***29.9***	38.4	36.1	37.5	37.3
npv	87.1	***87.9***	62.1	80.9	***85.8***

Baseline predictor performance on the unbalanced compendium 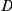. Values are compared with those for the typed predictors in Table 3 and set in italic when higher. If the difference is significant (two sided paired 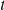-test, 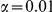) the entry is set in bold.

## Discussion

Recently, van't Veer and Bernards [41] claimed that the intrinsic breast cancer subtypes do not contain additional information for determining a patient's prognosis. They furthermore state that their value has been surpassed by that of prognostic gene-expression signatures such as the 70-gene signature, however, without quantifying these claims. In the current paper, we presented a framework for building and quantifying the performance of typed and untyped predictors, inspired by the protocol proposed by Wessels et al. [17]. Our results show that the subtype distribution of the training data has a considerable impact on the behavior of a predictor and we provide strong evidence that event prediction can be improved by exploiting subtype information. The highest performance is obtained by partitioning the samples into separate basal and Her2 groups, while using a combined luminal group.

These results are in line with improved predictive power that was also reported using an intrinsic gene list (IGL) approach by Parker et al. [8], which suggests a standardized gene set (PAM50) for subtype identification and event prediction. However, they only compare their subtype predictor with models based on standard clinicopathological parameters, such as estrogen receptor status and tumor size, and not with an untyped gene expression based predictor. The module-driven approach of Desmedt et al. [4] has also been used to combine subtype-specific predictors in a fuzzy way with promising results [9]. Although comprehensive, the latter work does not address influential factors like unequal class distributions or differences in the number of samples per subtype and presents its case for a single model, using a single partitioning scheme.

The module-driven approach was selected over the more common intrinsic gene list approach of Perou et al. [3] because of favorable stability properties, which are extensively addressed in [42]. We stress that even though the exact method used to generate subtype information is of interest, it is *not* the primary concern of this paper, as here we are mainly interested in how typed and untyped prediction can be properly compared given the various forms of imbalance.

### Sample size

As previously observed, stratification by subtype is accompanied by a sharp decrease in the number of samples available for predictor construction. Pairing typed predictors with untyped predictors offers the possibility to separately evaluate the influence of sample size and subtype information on classification performance. Our protocol incorporates two alternate views on sample size. Typed partitioning schemes involve multiple predictors, each targeting a specific subset of the entire sample set. Each typed predictor is paired with an untyped predictor, the construction of which involves an identical number of samples as for the typed predictor but with a subtype distribution that has been randomized such that it reflects the subtype distribution of the compendium. The advantage of matching sample size is that if subtyping would have no added value, paired typed and untyped predictors are expected to yield similar performance. Another view is provided by the comparison of typed predictors with the untyped baseline predictor in terms of overall performance. Prior to partitioning, all training sets are equally large. Hence, both typed and baseline predictor schemes involve the same total number of samples. According to both views typed predictors consistently outperform their untyped counterparts.

The potential to increase classification performance for breast cancer event prediction by combining data sets was recently addressed by Van Vliet et al. [12] which identified sample size as an important factor. In addition, it was observed that the performance on ER negative samples was much lower than achieved on ER positive samples, which matches well with the fact that the former group is substantially smaller than the latter. However, our work shows that when sample size is carefully controlled, performance differences between subtypes persist and cannot be ascribed solely to differences in sample size. For instance, basal samples, which are predominantly ER negative, appear an intrinsically more difficult set of samples to classify than Her2 samples.

### Class imbalance

We performed an analysis on a set of balanced and unbalanced compendia by which we show that typed predictors consistently outperform their untyped counterparts. Especially the balanced scenario shows the potential of typed predictors. In an unbalanced setting, however, it may be more challenging to exploit subtype information for various reasons. Typed schemes attempt to increase overall performance by predictors that perform well for all distinct parts. Such a strategy is not necessarily optimal in an unbalanced setting, as a predictor can be associated with a poor performance over all parts separately, yet can still give a reasonable overall performance over the union of these parts. This phenomenon is intimately related to the negative-positive class ratio and is perhaps easiest explained via the balanced accuracy rate (*bar*).

The *bar* is defined as the average of the sensitivity and specificity, that is, 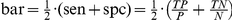, where 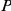 and 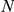 denote the number of positive and negative samples, respectively, and 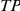 and 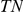 denote the true-positive and true-negative assignments made by a predictor. The *bar* score can be highly sensitive to the negative-positive class ratio in a subtle way. This becomes clear when rewriting the *bar* as a weighted accuracy measure
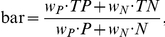
with weights 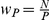 for the positive instances and 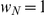 for the negative instances. Depending on the negative-positive class ratio, an error on a positive case is weighted differently from an error on a negative case. Hence, given the different negative-positive class ratios for different subtypes and for the whole compendium (Table 1), the same errors are weighted differently in the unbalanced compendium. For instance, the negative class is strongly overrepresented in the lumA subtype. In terms of *bar* the misclassification of a positive example in this case is extremely costly, as expressed by a *bar* of merely 51.8% in Table 4. The overall *bar*, however, weighs its errors very differently which results in a more optimistic *bar* of 65.1%. The latter example indicates the importance of proper stratification when comparing performances between groups.

In conclusion, we have presented a novel experimental protocol that allows for a proper comparison between typed and untyped predictors. We performed a comprehensive analysis of our methodology on a large breast cancer compendium and presented an analysis for balanced and unbalanced scenarios, which clearly reveal the potential of typed prediction. In both scenarios the highest overall performance was obtained by a typed partition which had separate Her2 and basal groups, while using a combined luminal group. In the balanced scenario it was observed that certain subtypes appear intrinsically more challenging as performance rates differ between subtypes. In an unbalanced setting it can be more difficult to exploit subtype information as the performance of certain subtypes can dominate overall performance. In addition, in such a scenario comparisons between predictors can be obscured by differences in sample size or class distribution. In our protocol sample size, class and subtype distributions are carefully controlled, which combined with the systematic pooling steps offers a rich view on the value of subtypes for event prediction.

## Supporting Information

Supporting Information S1
**Overview of the 892 samples comprising the compendium used for event prediction.** The column *CEL* indicates the accession number under which the corresponding expression data can be found for each individual sample. Entries starting with G refer to GEO accession numbers, while entries starting with E indicate ArrayExpress accession numbers. The column *t.dmfs* indicates distant metastasis free survival (in years), while the column *e.dmfs* indicates if a patient had an event i.e. a distant metastasis (1) or not (0). Finally, the last column indicates the class label for each sample (Good : *t.dmfs* 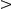
5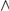 *e.dmfs* = 0, Poor : *t.dmfs* 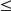
5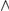 *e.dmfs* = 1).(PDF)

Supporting Information S2
**Additional classification results in which the ranking strategy, the predictor, and cross-validation scheme, respectively, have been altered compared to the setup corresponding to** **Figure 5** **.** The ranking strategy was altered from a ranking by moderated-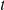 statistics to a ranking by signal-to-noise-ratio statistics (SNR). In addition, the nearest centroid (NC) predictor was replaced by the random forest (RF) predictor [40], which is a highly non-linear predictor. Finally, the 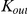 = 10-fold cross-validation strategy was changed to 3-fold cross-validation, 5-fold cross-validation, and leave-one-out cross-validation (LOOCV), respectively.(PDF)

Supporting Information S3
**Complete set of performances tables (similar to** **Tables 2** **and** **3** **of the main text) corresponding to** **Figure 5** **.** Each table provides a performance overview per elementary subtype: typed (tp) versus untyped (un) predictors, for a given partition, which is stated in the caption. The highest value for a paired typed and untyped performance measure is set in italic. If the difference is significant (two sided paired 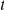-test, 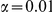) the entry is set in bold.(PDF)
