# Supplementary material for: An Evaluation Protocol for Subtype-Specific Breast Cancer Event Prediction
Source: PLoS One. 2011 Jul 8;6(7):e21681. doi: 10.1371/journal.pone.0021681 (PMC3132736; doi:10.1371/journal.pone.0021681)
Supplement: Supporting Information S1 — Overview of the 892 samples comprising the compendium used for event prediction. The column CEL indicates the accession number under which the corresponding expression data can be found for each individual sample. Entries starting with G refer to GEO accession numbers, while entries starting with E indicate ArrayExpress accession numbers. The column t.dmfs indicates distant metastasis free survival (in years), while the column e.dmfs indicates if a patient had an event i.e. a distant metastasis (1) or not (0). Finally, the last column indicates the class label for each sample (Good : t.dmfs 5 e.dmfs = 0, Poor : t.dmfs 5 e.dmfs = 1). (PDF) [file pone.0021681.s001.pdf]

Supporting Information S1 - An Evaluation Protocol for Subtype-Specific Breast Cancer Event Prediction

Herman MJ Sontrop[1], Wim FJ Verhaegh[1], Marcel JT Reinders[2,4], Perry D Moerland[3,4,*]

1 Molecular Diagnostics Department, Philips Research, High Tech Campus 11, 5656 AE Eindhoven, The Netherlands

2 Delft Bioinformatics Lab, Delft University of Technology, Mekelweg 4, 2628 CD Delft, The Netherlands

3 Bioinformatics Laboratory, Department of Clinical Epidemiology, Biostatistics, and Bioinformatics, Academic Medical Center, Meibergdreef 9, 1105 AZ Amsterdam, The Netherlands

4 Netherlands Bioinformatics Centre, Geert Grooteplein 28, 6525 GA Nijmegen, The Netherlands

∗ E-mail: p.d.moerland@amc.uva.nl

Table S1. CEL file and class label overview

Count	CEL	t.dmfs	e.dmfs	class label
1	GSM177885.CEL.gz	1.98	1	Poor
2	GSM177886.CEL.gz	18.06	0	Good
3	GSM177887.CEL.gz	1.44	1	Poor
4	GSM177890.CEL.gz	17.83	0	Good
5	GSM177891.CEL.gz	16.29	0	Good
6	GSM177893.CEL.gz	16.46	0	Good
7	GSM177894.CEL.gz	3.38	1	Poor
8	GSM177895.CEL.gz	3.11	1	Poor
9	GSM177896.CEL.gz	16.39	0	Good
10	GSM177897.CEL.gz	17.12	0	Good
11	GSM177899.CEL.gz	1.89	1	Poor
12	GSM177900.CEL.gz	2.72	1	Poor
13	GSM177901.CEL.gz	1.19	1	Poor
14	GSM177902.CEL.gz	4.78	1	Poor
15	GSM177904.CEL.gz	15.6	0	Good
16	GSM177905.CEL.gz	15.95	0	Good
17	GSM177906.CEL.gz	15.85	0	Good
18	GSM177907.CEL.gz	15.51	0	Good
19	GSM177909.CEL.gz	3.21	1	Poor
20	GSM177910.CEL.gz	14	0	Good
21	GSM177911.CEL.gz	15.16	0	Good
22	GSM177912.CEL.gz	15.26	0	Good
23	GSM177913.CEL.gz	15.12	0	Good
24	GSM177914.CEL.gz	14.74	0	Good
25	GSM177915.CEL.gz	12.81	0	Good
26	GSM177916.CEL.gz	12.75	0	Good
27	GSM177917.CEL.gz	11.58	0	Good
28	GSM177918.CEL.gz	2.49	1	Poor
29	GSM177919.CEL.gz	12.25	0	Good
30	GSM177920.CEL.gz	3.3	1	Poor
31	GSM177921.CEL.gz	19.33	0	Good
32	GSM177923.CEL.gz	14.68	0	Good

Continued on next page

Table S1 – continued from previous page

Count	CEL	t.dmfs	e.dmfs	class label
33	GSM177924.CEL.gz	16.29	0	Good
34	GSM177925.CEL.gz	15.48	0	Good
35	GSM177926.CEL.gz	1.45	1	Poor
36	GSM177927.CEL.gz	2.18	1	Poor
37	GSM177928.CEL.gz	15.58	0	Good
38	GSM177929.CEL.gz	2.8	1	Poor
39	GSM177930.CEL.gz	13.13	0	Good
40	GSM177932.CEL.gz	10.35	0	Good
41	GSM177933.CEL.gz	14.54	0	Good
42	GSM177934.CEL.gz	15.56	0	Good
43	GSM177935.CEL.gz	1.15	1	Poor
44	GSM177936.CEL.gz	15.38	0	Good
45	GSM177939.CEL.gz	12.85	0	Good
46	GSM177940.CEL.gz	14.35	0	Good
47	GSM177941.CEL.gz	13.98	0	Good
48	GSM177942.CEL.gz	7.7	0	Good
49	GSM177943.CEL.gz	9.03	0	Good
50	GSM177944.CEL.gz	9.74	0	Good
51	GSM177945.CEL.gz	14.88	0	Good
52	GSM177946.CEL.gz	14.32	0	Good
53	GSM177947.CEL.gz	14.56	0	Good
54	GSM177948.CEL.gz	13.49	0	Good
55	GSM177949.CEL.gz	13.73	0	Good
56	GSM177950.CEL.gz	12.72	0	Good
57	GSM177951.CEL.gz	9.07	0	Good
58	GSM177952.CEL.gz	13.32	0	Good
59	GSM177953.CEL.gz	4.97	1	Poor
60	GSM177954.CEL.gz	2.2	1	Poor
61	GSM177955.CEL.gz	5.77	0	Good
62	GSM177956.CEL.gz	1.45	1	Poor
63	GSM177957.CEL.gz	13.23	0	Good
64	GSM177958.CEL.gz	4.72	1	Poor
65	GSM177959.CEL.gz	12.93	0	Good
66	GSM177960.CEL.gz	3.45	1	Poor
67	GSM177965.CEL.gz	10.68	0	Good
68	GSM177967.CEL.gz	9.71	0	Good
69	GSM177968.CEL.gz	10.19	0	Good
70	GSM177969.CEL.gz	10.33	0	Good
71	GSM177970.CEL.gz	9.02	0	Good
72	GSM177971.CEL.gz	10.41	0	Good
73	GSM177973.CEL.gz	1.78	1	Poor
74	GSM177974.CEL.gz	13.57	0	Good
75	GSM177975.CEL.gz	4.64	1	Poor
76	GSM177976.CEL.gz	12.62	0	Good
77	GSM177977.CEL.gz	13.31	0	Good
78	GSM177978.CEL.gz	12.83	0	Good
79	GSM177980.CEL.gz	12.6	0	Good

Continued on next page

Table S1 – continued from previous page

Count	CEL	t.dmfs	e.dmfs	class label
80	GSM177981.CEL.gz	3.05	1	Poor
81	GSM177983.CEL.gz	4.74	1	Poor
82	GSM177984.CEL.gz	11.86	0	Good
83	GSM177985.CEL.gz	11.93	0	Good
84	GSM177986.CEL.gz	11.46	0	Good
85	GSM177987.CEL.gz	8.74	0	Good
86	GSM177988.CEL.gz	7.32	0	Good
87	GSM177989.CEL.gz	2	1	Poor
88	GSM177991.CEL.gz	5.26	0	Good
89	GSM177992.CEL.gz	7.25	0	Good
90	GSM177993.CEL.gz	0.74	1	Poor
91	GSM177995.CEL.gz	12.52	0	Good
92	GSM177996.CEL.gz	12.43	0	Good
93	GSM177997.CEL.gz	13.79	0	Good
94	GSM177999.CEL.gz	1.08	1	Poor
95	GSM178002.CEL.gz	11.19	0	Good
96	GSM178004.CEL.gz	14.35	0	Good
97	GSM178005.CEL.gz	14.81	0	Good
98	GSM178006.CEL.gz	11.72	0	Good
99	GSM178007.CEL.gz	14.68	0	Good
100	GSM178008.CEL.gz	10.86	0	Good
101	GSM178009.CEL.gz	0.79	1	Poor
102	GSM178013.CEL.gz	17.1	0	Good
103	GSM178014.CEL.gz	16.88	0	Good
104	GSM178015.CEL.gz	16.8	0	Good
105	GSM178017.CEL.gz	13.64	0	Good
106	GSM178018.CEL.gz	4.67	1	Poor
107	GSM178019.CEL.gz	13.74	0	Good
108	GSM178020.CEL.gz	2.41	1	Poor
109	GSM178001.CEL.gz	6.98	0	Good
110	GSM178010.CEL.gz	17.25	0	Good
111	GSM178011.CEL.gz	17.53	0	Good
112	GSM178012.CEL.gz	15.57	0	Good
113	GSM178021.CEL.gz	23.87	0	Good
114	GSM178022.CEL.gz	12.8	0	Good
115	GSM178023.CEL.gz	19.02	0	Good
116	GSM178024.CEL.gz	16.81	0	Good
117	GSM178025.CEL.gz	2.21	1	Poor
118	GSM178026.CEL.gz	16.21	0	Good
119	GSM178027.CEL.gz	16.19	0	Good
120	GSM178029.CEL.gz	16.05	0	Good
121	GSM178031.CEL.gz	15.83	0	Good
122	GSM178032.CEL.gz	15.83	0	Good
123	GSM178033.CEL.gz	15.68	0	Good
124	GSM178034.CEL.gz	15.65	0	Good
125	GSM178035.CEL.gz	15.49	0	Good
126	GSM178036.CEL.gz	0.34	1	Poor

Continued on next page

Table S1 – continued from previous page

Count	CEL	t.dmfs	e.dmfs	class label
127	GSM178037.CEL.gz	15.56	0	Good
128	GSM178038.CEL.gz	4.77	1	Poor
129	GSM178040.CEL.gz	15.35	0	Good
130	GSM178041.CEL.gz	15.8	0	Good
131	GSM178042.CEL.gz	15.57	0	Good
132	GSM178043.CEL.gz	15.15	0	Good
133	GSM178044.CEL.gz	15.21	0	Good
134	GSM178045.CEL.gz	17.4	0	Good
135	GSM178046.CEL.gz	17.25	0	Good
136	GSM178048.CEL.gz	17.21	0	Good
137	GSM178050.CEL.gz	16.45	0	Good
138	GSM178052.CEL.gz	17.07	0	Good
139	GSM178053.CEL.gz	24.95	0	Good
140	GSM178055.CEL.gz	10.72	0	Good
141	GSM178056.CEL.gz	14.19	0	Good
142	GSM178057.CEL.gz	10.56	0	Good
143	GSM178058.CEL.gz	14.13	0	Good
144	GSM178059.CEL.gz	13	0	Good
145	GSM178060.CEL.gz	2.18	1	Poor
146	GSM178061.CEL.gz	4.38	1	Poor
147	GSM178062.CEL.gz	13.8	0	Good
148	GSM178063.CEL.gz	13.35	0	Good
149	GSM178064.CEL.gz	12.48	0	Good
150	GSM178065.CEL.gz	12.29	0	Good
151	GSM178066.CEL.gz	11.77	0	Good
152	GSM178067.CEL.gz	13.33	0	Good
153	GSM178068.CEL.gz	11.45	0	Good
154	GSM178070.CEL.gz	11.1	0	Good
155	GSM178071.CEL.gz	10.36	0	Good
156	GSM178072.CEL.gz	9.57	0	Good
157	GSM178073.CEL.gz	7.02	0	Good
158	GSM178075.CEL.gz	7.89	0	Good
159	GSM178076.CEL.gz	14.07	0	Good
160	GSM178077.CEL.gz	19.24	0	Good
161	GSM178078.CEL.gz	15.54	0	Good
162	GSM178079.CEL.gz	1.11	1	Poor
163	GSM178080.CEL.gz	6.1	0	Good
164	GSM178081.CEL.gz	7.46	0	Good
165	GSM50034.CEL.gz	7.25	0	Good
166	GSM50035.CEL.gz	1.84	1	Poor
167	GSM50036.CEL.gz	9.14	0	Good
168	GSM50037.CEL.gz	6.86	0	Good
169	GSM50038.CEL.gz	6.47	0	Good
170	GSM50039.CEL.gz	7.08	0	Good
171	GSM50040.CEL.gz	7.57	0	Good
172	GSM50043.CEL.gz	5.23	0	Good
173	GSM50044.CEL.gz	5.62	0	Good

Continued on next page

Table S1 – continued from previous page

Count	CEL	t.dmfs	e.dmfs	class label
174	GSM50045.CEL.gz	10.75	0	Good
175	GSM50046.CEL.gz	8.04	0	Good
176	GSM50047.CEL.gz	5.41	0	Good
177	GSM50048.CEL.gz	6.07	0	Good
178	GSM50049.CEL.gz	5.5	0	Good
179	GSM50059.CEL.gz	7.63	0	Good
180	GSM50060.CEL.gz	3.47	1	Poor
181	GSM50061.CEL.gz	3.96	1	Poor
182	GSM50062.CEL.gz	3.07	1	Poor
183	GSM50063.CEL.gz	3.11	1	Poor
184	GSM50064.CEL.gz	0.94	1	Poor
185	GSM50065.CEL.gz	5.81	0	Good
186	GSM50066.CEL.gz	1.24	1	Poor
187	GSM50067.CEL.gz	0.77	1	Poor
188	GSM50068.CEL.gz	3.48	1	Poor
189	GSM50069.CEL.gz	3.83	1	Poor
190	GSM50070.CEL.gz	3.36	1	Poor
191	GSM50071.CEL.gz	5.82	0	Good
192	GSM50072.CEL.gz	10.74	0	Good
193	GSM50073.CEL.gz	7.25	0	Good
194	GSM50074.CEL.gz	7.32	0	Good
195	GSM50075.CEL.gz	7.32	0	Good
196	GSM50078.CEL.gz	6.98	0	Good
197	GSM50079.CEL.gz	6.52	0	Good
198	GSM50080.CEL.gz	7.25	0	Good
199	GSM50081.CEL.gz	5.07	0	Good
200	GSM50082.CEL.gz	7.11	0	Good
201	GSM50083.CEL.gz	7.28	0	Good
202	GSM50084.CEL.gz	6.4	0	Good
203	GSM50085.CEL.gz	6.5	0	Good
204	GSM50086.CEL.gz	8.31	0	Good
205	GSM50087.CEL.gz	8.31	0	Good
206	GSM50089.CEL.gz	8.74	0	Good
207	GSM50090.CEL.gz	6.79	0	Good
208	GSM50091.CEL.gz	7.19	0	Good
209	GSM50092.CEL.gz	6.45	0	Good
210	GSM50093.CEL.gz	7.86	0	Good
211	GSM50094.CEL.gz	3.23	1	Poor
212	GSM50096.CEL.gz	1.38	1	Poor
213	GSM50098.CEL.gz	2.57	1	Poor
214	GSM50099.CEL.gz	1.3	1	Poor
215	GSM50102.CEL.gz	0.67	1	Poor
216	GSM50103.CEL.gz	1.84	1	Poor
217	GSM50105.CEL.gz	7.19	0	Good
218	GSM50106.CEL.gz	1.57	1	Poor
219	GSM50107.CEL.gz	6.21	0	Good
220	GSM50110.CEL.gz	5.19	0	Good

Continued on next page

Table S1 – continued from previous page

Count	CEL	t.dmfs	e.dmfs	class label
221	GSM50111.CEL.gz	7.3	0	Good
222	GSM50118.CEL.gz	6.28	0	Good
223	GSM50122.CEL.gz	3.24	1	Poor
224	GSM50127.CEL.gz	5.24	0	Good
225	GSM50131.CEL.gz	4.59	1	Poor
226	GSM65875.CEL.gz	8.94	0	Good
227	GSM65829.CEL.gz	11.41	0	Good
228	GSM65830.CEL.gz	13.34	0	Good
229	GSM65876.CEL.gz	5.24	0	Good
230	GSM65820.CEL.gz	14.53	0	Good
231	GSM65831.CEL.gz	13.78	0	Good
232	GSM65832.CEL.gz	12.92	0	Good
233	GSM65880.CEL.gz	7.7	0	Good
234	GSM65821.CEL.gz	11.18	0	Good
235	GSM65835.CEL.gz	1.54	1	Poor
236	GSM65840.CEL.gz	12.64	0	Good
237	GSM65841.CEL.gz	10.56	0	Good
238	GSM65844.CEL.gz	0.61	1	Poor
239	GSM65847.CEL.gz	2.92	1	Poor
240	GSM65848.CEL.gz	12.79	0	Good
241	GSM65849.CEL.gz	2.89	1	Poor
242	GSM65850.CEL.gz	12.42	0	Good
243	GSM65851.CEL.gz	12.47	0	Good
244	GSM65853.CEL.gz	10.72	0	Good
245	GSM65854.CEL.gz	12.27	0	Good
246	GSM65856.CEL.gz	9.82	0	Good
247	GSM65858.CEL.gz	12.04	0	Good
248	GSM65859.CEL.gz	12.32	0	Good
249	GSM65860.CEL.gz	12.25	0	Good
250	GSM65823.CEL.gz	14.19	0	Good
251	GSM65861.CEL.gz	2.64	1	Poor
252	GSM65864.CEL.gz	10.04	0	Good
253	GSM65865.CEL.gz	12.06	0	Good
254	GSM65866.CEL.gz	11.17	0	Good
255	GSM65867.CEL.gz	11.56	0	Good
256	GSM65868.CEL.gz	3.01	1	Poor
257	GSM65869.CEL.gz	2.63	1	Poor
258	GSM65825.CEL.gz	13.96	0	Good
259	GSM65827.CEL.gz	13.78	0	Good
260	GSM65807.CEL.gz	9.24	0	Good
261	GSM65810.CEL.gz	8.83	0	Good
262	GSM65819.CEL.gz	11.41	0	Good
263	GSM65362.CEL.gz	7.58	0	Good
264	GSM150944.CEL.gz	7.74	0	Good
265	GSM150946.CEL.gz	3.72	1	Poor
266	GSM150947.CEL.gz	2.29	1	Poor
267	GSM65361.CEL.gz	7.36	0	Good

Continued on next page

Table S1 – continued from previous page

Count	CEL	t.dmfs	e.dmfs	class label
268	GSM150948.CEL.gz	7.28	0	Good
269	GSM65358.CEL.gz	5.58	0	Good
270	GSM150949.CEL.gz	7.79	0	Good
271	GSM150950.CEL.gz	5.11	0	Good
272	GSM150951.CEL.gz	5.16	0	Good
273	GSM150953.CEL.gz	8	0	Good
274	GSM150954.CEL.gz	6.25	0	Good
275	GSM150956.CEL.gz	3.1	1	Poor
276	GSM150957.CEL.gz	6.93	0	Good
277	GSM150958.CEL.gz	7.19	0	Good
278	GSM150959.CEL.gz	7.04	0	Good
279	GSM150960.CEL.gz	1.98	1	Poor
280	GSM150961.CEL.gz	1.96	1	Poor
281	GSM150963.CEL.gz	7.92	0	Good
282	GSM150965.CEL.gz	6.86	0	Good
283	GSM65360.CEL.gz	7.01	0	Good
284	GSM65359.CEL.gz	5.74	0	Good
285	GSM150966.CEL.gz	5.97	0	Good
286	GSM150969.CEL.gz	3.63	1	Poor
287	GSM150973.CEL.gz	5.36	0	Good
288	GSM150974.CEL.gz	3.78	1	Poor
289	GSM150977.CEL.gz	5.86	0	Good
290	GSM65355.CEL.gz	5.16	0	Good
291	GSM150978.CEL.gz	5.18	0	Good
292	GSM150979.CEL.gz	5.65	0	Good
293	GSM65357.CEL.gz	5.48	0	Good
294	GSM65370.CEL.gz	1.74	1	Poor
295	GSM65367.CEL.gz	9.88	0	Good
296	GSM150983.CEL.gz	10.49	0	Good
297	GSM150984.CEL.gz	9.35	0	Good
298	GSM150985.CEL.gz	10.58	0	Good
299	GSM150986.CEL.gz	10.16	0	Good
300	GSM150987.CEL.gz	3.77	1	Poor
301	GSM65374.CEL.gz	4.53	1	Poor
302	GSM65356.CEL.gz	5.32	0	Good
303	GSM150989.CEL.gz	5.19	0	Good
304	GSM150990.CEL.gz	9.8	0	Good
305	GSM150991.CEL.gz	12.75	0	Good
306	GSM150992.CEL.gz	9.78	0	Good
307	GSM150993.CEL.gz	9.58	0	Good
308	GSM65371.CEL.gz	1.55	1	Poor
309	GSM65372.CEL.gz	2.57	1	Poor
310	GSM65368.CEL.gz	10.29	0	Good
311	GSM65366.CEL.gz	9.41	0	Good
312	GSM150994.CEL.gz	11.05	0	Good
313	GSM150995.CEL.gz	10.38	0	Good
314	GSM65363.CEL.gz	8.59	0	Good

Continued on next page

Table S1 – continued from previous page

Count	CEL	t.dmfs	e.dmfs	class label
315	GSM150996.CEL.gz	10.01	0	Good
316	GSM150997.CEL.gz	9.88	0	Good
317	GSM150998.CEL.gz	2.67	1	Poor
318	GSM150999.CEL.gz	3.54	1	Poor
319	GSM65379.CEL.gz	8.1	0	Good
320	GSM65365.CEL.gz	8.88	0	Good
321	GSM65364.CEL.gz	8.81	0	Good
322	GSM151001.CEL.gz	1.35	1	Poor
323	GSM151002.CEL.gz	6.04	0	Good
324	GSM151003.CEL.gz	5.46	0	Good
325	GSM151005.CEL.gz	9.43	0	Good
326	GSM151008.CEL.gz	8.24	0	Good
327	GSM151009.CEL.gz	8.09	0	Good
328	GSM151010.CEL.gz	8	0	Good
329	GSM151011.CEL.gz	8.11	0	Good
330	E-TABM-158-raw-cel-1639356804.CEL	11.32	0	Good
331	E-TABM-158-raw-cel-1639356292.CEL	0.34	1	Poor
332	E-TABM-158-raw-cel-1639357692.CEL	5.61	0	Good
333	E-TABM-158-raw-cel-1639356930.CEL	7.56	0	Good
334	E-TABM-158-raw-cel-1639357226.CEL	11.35	0	Good
335	E-TABM-158-raw-cel-1639357976.CEL	0.31	1	Poor
336	E-TABM-158-raw-cel-1639356033.CEL	6.02	0	Good
337	E-TABM-158-raw-cel-1639356992.CEL	0.42	1	Poor
338	E-TABM-158-raw-cel-1639356073.CEL	8.67	0	Good
339	E-TABM-158-raw-cel-1639357076.CEL	5.64	0	Good
340	E-TABM-158-raw-cel-1639358268.CEL	10.98	0	Good
341	E-TABM-158-raw-cel-1639357400.CEL	6.76	0	Good
342	E-TABM-158-raw-cel-1639357996.CEL	10.72	0	Good
343	E-TABM-158-raw-cel-1639356758.CEL	0.67	1	Poor
344	E-TABM-158-raw-cel-1639355965.CEL	9.98	0	Good
345	E-TABM-158-raw-cel-1639358352.CEL	4.33	1	Poor
346	E-TABM-158-raw-cel-1639358228.CEL	1.86	1	Poor
347	E-TABM-158-raw-cel-1639356204.CEL	7.86	0	Good
348	E-TABM-158-raw-cel-1639356420.CEL	5.28	0	Good
349	E-TABM-158-raw-cel-1639357380.CEL	5.63	0	Good
350	E-TABM-158-raw-cel-1639357184.CEL	10.36	0	Good
351	E-TABM-158-raw-cel-1639358016.CEL	7.47	0	Good
352	E-TABM-158-raw-cel-1639355793.CEL	0	1	Poor
353	E-TABM-158-raw-cel-1639358060.CEL	11.28	0	Good
354	E-TABM-158-raw-cel-1639356228.CEL	5.98	0	Good
355	E-TABM-158-raw-cel-1639356588.CEL	7.66	0	Good
356	E-TABM-158-raw-cel-1639357248.CEL	10.45	0	Good
357	E-TABM-158-raw-cel-1639355813.CEL	8.01	0	Good
358	E-TABM-158-raw-cel-1639357034.CEL	10.98	0	Good
359	E-TABM-158-raw-cel-1639356612.CEL	5.79	0	Good
360	E-TABM-158-raw-cel-1639356886.CEL	9.6	0	Good
361	E-TABM-158-raw-cel-1639357848.CEL	9.14	0	Good

Continued on next page

Table S1 – continued from previous page

Count	CEL	t.dmfs	e.dmfs	class label
362	E-TABM-158-raw-cel-1639355709.CEL	6.02	0	Good
363	E-TABM-158-raw-cel-1639356272.CEL	11.78	0	Good
364	E-TABM-158-raw-cel-1639357556.CEL	7.9	0	Good
365	E-TABM-158-raw-cel-1639356097.CEL	10.3	0	Good
366	E-TABM-158-raw-cel-1639358208.CEL	10.2	0	Good
367	E-TABM-158-raw-cel-1639356441.CEL	8.18	0	Good
368	E-TABM-158-raw-cel-1639357014.CEL	6.84	0	Good
369	E-TABM-158-raw-cel-1639357668.CEL	8.51	0	Good
370	E-TABM-158-raw-cel-1639356824.CEL	4.04	1	Poor
371	E-TABM-158-raw-cel-1639357140.CEL	9.71	0	Good
372	E-TABM-158-raw-cel-1639356312.CEL	2.47	1	Poor
373	E-TABM-158-raw-cel-1639357624.CEL	8.38	0	Good
374	E-TABM-158-raw-cel-1639356184.CEL	2.88	1	Poor
375	E-TABM-158-raw-cel-1639357580.CEL	11.96	0	Good
376	E-TABM-158-raw-cel-1639356676.CEL	9.25	0	Good
377	E-TABM-158-raw-cel-1639356548.CEL	1.15	1	Poor
378	E-TABM-158-raw-cel-1639356248.CEL	0	1	Poor
379	E-TABM-158-raw-cel-1639357956.CEL	9.01	0	Good
380	E-TABM-158-raw-cel-1639357056.CEL	8.08	0	Good
381	E-TABM-158-raw-cel-1639357756.CEL	6.36	0	Good
382	E-TABM-158-raw-cel-1639356524.CEL	5.57	0	Good
383	E-TABM-158-raw-cel-1639356464.CEL	9.47	0	Good
384	E-TABM-158-raw-cel-1639356908.CEL	0	1	Poor
385	E-TABM-158-raw-cel-1639356356.CEL	12.87	0	Good
386	E-TABM-158-raw-cel-1639357118.CEL	11.35	0	Good
387	E-TABM-158-raw-cel-1639356780.CEL	2.71	1	Poor
388	E-TABM-158-raw-cel-1639356053.CEL	10.09	0	Good
389	E-TABM-158-raw-cel-1639357336.CEL	8.96	0	Good
390	E-TABM-158-raw-cel-1639356568.CEL	6.73	0	Good
391	E-TABM-158-raw-cel-1639357444.CEL	0.67	1	Poor
392	E-TABM-158-raw-cel-1639358144.CEL	8.91	0	Good
393	E-TABM-158-raw-cel-1639358440.CEL	13.75	0	Good
394	E-TABM-158-raw-cel-1639355773.CEL	1.32	1	Poor
395	E-TABM-158-raw-cel-1639355943.CEL	9.6	0	Good
396	E-TABM-158-raw-cel-1639357780.CEL	10.34	0	Good
397	E-TABM-158-raw-cel-1639358460.CEL	5.7	0	Good
398	E-TABM-158-raw-cel-1639356696.CEL	12.78	0	Good
399	E-TABM-158-raw-cel-1639355985.CEL	10.31	0	Good
400	E-TABM-158-raw-cel-1639358396.CEL	11.24	0	Good
401	E-TABM-158-raw-cel-1639357162.CEL	3.51	1	Poor
402	E-TABM-158-raw-cel-1639355921.CEL	9.8	0	Good
403	E-TABM-158-raw-cel-1639357424.CEL	11.1	0	Good
404	E-TABM-158-raw-cel-1639357468.CEL	2.27	1	Poor
405	E-TABM-158-raw-cel-1639356736.CEL	0	1	Poor
406	E-TABM-158-raw-cel-1639356504.CEL	9.1	0	Good
407	E-TABM-158-raw-cel-1639355859.CEL	3.62	1	Poor
408	E-TABM-158-raw-cel-1639358124.CEL	10.26	0	Good

Continued on next page

Table S1 – continued from previous page

Count	CEL	t.dmfs	e.dmfs	class label
409	E-TABM-158-raw-cel-1639357488.CEL	0	1	Poor
410	E-TABM-158-raw-cel-1639355750.CEL	1.53	1	Poor
411	E-TABM-158-raw-cel-1639355837.CEL	6.12	0	Good
412	E-TABM-158-raw-cel-1639356716.CEL	8.53	0	Good
413	E-TABM-158-raw-cel-1639356652.CEL	6.94	0	Good
414	E-TABM-158-raw-cel-1639358292.CEL	2.15	1	Poor
415	E-TABM-158-raw-cel-1639358040.CEL	9.94	0	Good
416	GSM36793.CEL.gz	8.46	0	Good
417	GSM36796.CEL.gz	9.8	0	Good
418	GSM36797.CEL.gz	0.76	1	Poor
419	GSM36798.CEL.gz	8.8	0	Good
420	GSM36800.CEL.gz	3.09	1	Poor
421	GSM36801.CEL.gz	10.41	0	Good
422	GSM36834.CEL.gz	9.1	0	Good
423	GSM36835.CEL.gz	1.13	1	Poor
424	GSM36836.CEL.gz	8.22	0	Good
425	GSM36837.CEL.gz	11.38	0	Good
426	GSM36838.CEL.gz	2.8	1	Poor
427	GSM36839.CEL.gz	2.69	1	Poor
428	GSM36855.CEL.gz	10.69	0	Good
429	GSM36858.CEL.gz	1.17	1	Poor
430	GSM36859.CEL.gz	10.85	0	Good
431	GSM36860.CEL.gz	2.54	1	Poor
432	GSM36861.CEL.gz	12.91	0	Good
433	GSM36862.CEL.gz	2.09	1	Poor
434	GSM36870.CEL.gz	2.54	1	Poor
435	GSM36871.CEL.gz	7.04	0	Good
436	GSM36872.CEL.gz	0.6	1	Poor
437	GSM36873.CEL.gz	8.34	0	Good
438	GSM36874.CEL.gz	2.49	1	Poor
439	GSM36875.CEL.gz	0.62	1	Poor
440	GSM36876.CEL.gz	11.08	0	Good
441	GSM36877.CEL.gz	3.58	1	Poor
442	GSM36896.CEL.gz	8.52	0	Good
443	GSM36897.CEL.gz	2.05	1	Poor
444	GSM36899.CEL.gz	14.12	0	Good
445	GSM36900.CEL.gz	9.05	0	Good
446	GSM36902.CEL.gz	2.35	1	Poor
447	GSM36916.CEL.gz	10.44	0	Good
448	GSM36917.CEL.gz	12.03	0	Good
449	GSM36918.CEL.gz	0.91	1	Poor
450	GSM36919.CEL.gz	12.77	0	Good
451	GSM36920.CEL.gz	1.63	1	Poor
452	GSM36937.CEL.gz	0.91	1	Poor
453	GSM36938.CEL.gz	9	0	Good
454	GSM36939.CEL.gz	1.16	1	Poor
455	GSM36940.CEL.gz	11.16	0	Good

Continued on next page

Table S1 – continued from previous page

Count	CEL	t.dmfs	e.dmfs	class label
456	GSM36941.CEL.gz	0.74	1	Poor
457	GSM36942.CEL.gz	12.63	0	Good
458	GSM36943.CEL.gz	1.58	1	Poor
459	GSM36944.CEL.gz	9.32	0	Good
460	GSM36962.CEL.gz	9.46	0	Good
461	GSM36964.CEL.gz	0.98	1	Poor
462	GSM36965.CEL.gz	11.13	0	Good
463	GSM36966.CEL.gz	7.46	0	Good
464	GSM36967.CEL.gz	1.29	1	Poor
465	GSM36968.CEL.gz	12.67	0	Good
466	GSM36969.CEL.gz	2.64	1	Poor
467	GSM36987.CEL.gz	8.07	0	Good
468	GSM36989.CEL.gz	0.93	1	Poor
469	GSM36990.CEL.gz	11.91	0	Good
470	GSM36991.CEL.gz	9.47	0	Good
471	GSM36992.CEL.gz	8.67	0	Good
472	GSM36993.CEL.gz	8.16	0	Good
473	GSM36994.CEL.gz	0.19	1	Poor
474	GSM37017.CEL.gz	9.01	0	Good
475	GSM37018.CEL.gz	1.66	1	Poor
476	GSM37019.CEL.gz	12.14	0	Good
477	GSM37020.CEL.gz	4	1	Poor
478	GSM37021.CEL.gz	9.06	0	Good
479	GSM37022.CEL.gz	2.48	1	Poor
480	GSM37023.CEL.gz	1.23	1	Poor
481	GSM37025.CEL.gz	8.96	0	Good
482	GSM36879.CEL.gz	3.24	1	Poor
483	GSM36880.CEL.gz	8.09	0	Good
484	GSM36881.CEL.gz	0.91	1	Poor
485	GSM36882.CEL.gz	8.26	0	Good
486	GSM36885.CEL.gz	3.34	1	Poor
487	GSM36886.CEL.gz	7.82	0	Good
488	GSM36888.CEL.gz	2.36	1	Poor
489	GSM36891.CEL.gz	7.22	0	Good
490	GSM36903.CEL.gz	0.64	1	Poor
491	GSM36904.CEL.gz	8.19	0	Good
492	GSM36905.CEL.gz	1.42	1	Poor
493	GSM36906.CEL.gz	8.42	0	Good
494	GSM36907.CEL.gz	7.13	0	Good
495	GSM36908.CEL.gz	1.28	1	Poor
496	GSM36909.CEL.gz	7.89	0	Good
497	GSM36923.CEL.gz	1.94	1	Poor
498	GSM36925.CEL.gz	8.64	0	Good
499	GSM36927.CEL.gz	0.64	1	Poor
500	GSM36929.CEL.gz	7.72	0	Good
501	GSM36931.CEL.gz	3.04	1	Poor
502	GSM36946.CEL.gz	2.17	1	Poor

Continued on next page

Table S1 – continued from previous page

Count	CEL	t.dmfs	e.dmfs	class label
503	GSM36948.CEL.gz	7.25	0	Good
504	GSM36950.CEL.gz	2.33	1	Poor
505	GSM36953.CEL.gz	7	0	Good
506	GSM36955.CEL.gz	2.11	1	Poor
507	GSM36956.CEL.gz	2.66	1	Poor
508	GSM36957.CEL.gz	3.94	1	Poor
509	GSM36958.CEL.gz	9.27	0	Good
510	GSM36971.CEL.gz	1.56	1	Poor
511	GSM36973.CEL.gz	2.07	1	Poor
512	GSM36921.CEL.gz	8.96	0	Good
513	GSM36922.CEL.gz	9.43	0	Good
514	GSM36924.CEL.gz	2.44	1	Poor
515	GSM36926.CEL.gz	2.04	1	Poor
516	GSM36928.CEL.gz	1.89	1	Poor
517	GSM36898.CEL.gz	0.56	1	Poor
518	GSM36947.CEL.gz	1.31	1	Poor
519	GSM36949.CEL.gz	0.47	1	Poor
520	GSM36952.CEL.gz	1.08	1	Poor
521	GSM36954.CEL.gz	1.6	1	Poor
522	GSM36972.CEL.gz	3.09	1	Poor
523	GSM36974.CEL.gz	1.88	1	Poor
524	GSM36976.CEL.gz	3.2	1	Poor
525	GSM36996.CEL.gz	2.01	1	Poor
526	GSM36997.CEL.gz	3.64	1	Poor
527	GSM36998.CEL.gz	0.42	1	Poor
528	GSM36999.CEL.gz	3.11	1	Poor
529	GSM37001.CEL.gz	2.77	1	Poor
530	GSM37002.CEL.gz	1.3	1	Poor
531	GSM37003.CEL.gz	1.54	1	Poor
532	GSM37004.CEL.gz	2.91	1	Poor
533	GSM37026.CEL.gz	3.01	1	Poor
534	GSM37027.CEL.gz	1.44	1	Poor
535	GSM37028.CEL.gz	3.26	1	Poor
536	GSM37029.CEL.gz	3.96	1	Poor
537	GSM37030.CEL.gz	3.64	1	Poor
538	GSM37031.CEL.gz	1.5	1	Poor
539	GSM37035.CEL.gz	1.2	1	Poor
540	GSM37036.CEL.gz	1.26	1	Poor
541	GSM37037.CEL.gz	1.61	1	Poor
542	GSM37039.CEL.gz	4	1	Poor
543	GSM37040.CEL.gz	0.48	1	Poor
544	GSM37041.CEL.gz	0.77	1	Poor
545	GSM37042.CEL.gz	1.17	1	Poor
546	GSM37049.CEL.gz	2.43	1	Poor
547	GSM37050.CEL.gz	1.52	1	Poor
548	GSM37051.CEL.gz	1.58	1	Poor
549	GSM37052.CEL.gz	2.71	1	Poor

Continued on next page

Table S1 – continued from previous page

Count	CEL	t.dmfs	e.dmfs	class label
550	GSM37053.CEL.gz	2.71	1	Poor
551	GSM37054.CEL.gz	5.49	0	Good
552	GSM37056.CEL.gz	6.86	0	Good
553	GSM37059.CEL.gz	6.89	0	Good
554	GSM37060.CEL.gz	6.77	0	Good
555	GSM37061.CEL.gz	8.78	0	Good
556	GSM36777.CEL.gz	6.59	0	Good
557	GSM36787.CEL.gz	5.97	0	Good
558	GSM36789.CEL.gz	4.24	1	Poor
559	GSM36790.CEL.gz	8.93	0	Good
560	GSM36791.CEL.gz	6.59	0	Good
561	GSM36805.CEL.gz	8.33	0	Good
562	GSM36813.CEL.gz	4.09	1	Poor
563	GSM36826.CEL.gz	4.83	1	Poor
564	GSM36782.CEL.gz	5.51	0	Good
565	GSM36784.CEL.gz	4.78	1	Poor
566	GSM36786.CEL.gz	5.53	0	Good
567	GSM36788.CEL.gz	6.61	0	Good
568	GSM36799.CEL.gz	6.29	0	Good
569	GSM36802.CEL.gz	7.65	0	Good
570	GSM36803.CEL.gz	7.74	0	Good
571	GSM36804.CEL.gz	7.33	0	Good
572	GSM36806.CEL.gz	7.67	0	Good
573	GSM36807.CEL.gz	7.3	0	Good
574	GSM36812.CEL.gz	6.69	0	Good
575	GSM36817.CEL.gz	9.42	0	Good
576	GSM36819.CEL.gz	10.88	0	Good
577	GSM36823.CEL.gz	9.89	0	Good
578	GSM36825.CEL.gz	8.22	0	Good
579	GSM36828.CEL.gz	6.35	0	Good
580	GSM36830.CEL.gz	9.75	0	Good
581	GSM36832.CEL.gz	9.95	0	Good
582	GSM36842.CEL.gz	6.77	0	Good
583	GSM36843.CEL.gz	6.43	0	Good
584	GSM36851.CEL.gz	8.35	0	Good
585	GSM36854.CEL.gz	8.76	0	Good
586	GSM36857.CEL.gz	7.96	0	Good
587	GSM36960.CEL.gz	4.25	1	Poor
588	GSM36963.CEL.gz	5.03	0	Good
589	GSM36816.CEL.gz	8.13	0	Good
590	GSM36824.CEL.gz	7.18	0	Good
591	GSM36827.CEL.gz	12.96	0	Good
592	GSM36829.CEL.gz	13.07	0	Good
593	GSM36831.CEL.gz	7.28	0	Good
594	GSM36848.CEL.gz	8.71	0	Good
595	GSM36850.CEL.gz	10.05	0	Good
596	GSM36853.CEL.gz	8.73	0	Good

Continued on next page

Table S1 – continued from previous page

Count	CEL	t.dmfs	e.dmfs	class label
597	GSM36856.CEL.gz	7.13	0	Good
598	GSM36868.CEL.gz	10.25	0	Good
599	GSM36869.CEL.gz	10.51	0	Good
600	GSM36893.CEL.gz	10.31	0	Good
601	GSM36895.CEL.gz	7.99	0	Good
602	GSM36820.CEL.gz	11.79	0	Good
603	GSM36910.CEL.gz	10.43	0	Good
604	GSM36913.CEL.gz	9.08	0	Good
605	GSM36878.CEL.gz	8.19	0	Good
606	GSM36970.CEL.gz	9.69	0	Good
607	GSM36934.CEL.gz	14.22	0	Good
608	GSM36781.CEL.gz	12.22	0	Good
609	GSM36841.CEL.gz	11.2	0	Good
610	GSM36884.CEL.gz	10.74	0	Good
611	GSM36930.CEL.gz	6.98	0	Good
612	GSM37047.CEL.gz	8.99	0	Good
613	GSM36959.CEL.gz	10	0	Good
614	GSM36961.CEL.gz	12.71	0	Good
615	GSM36951.CEL.gz	8.66	0	Good
616	GSM37000.CEL.gz	11.39	0	Good
617	GSM36977.CEL.gz	10.31	0	Good
618	GSM36978.CEL.gz	12.34	0	Good
619	GSM36980.CEL.gz	10.17	0	Good
620	GSM36984.CEL.gz	11.5	0	Good
621	GSM36975.CEL.gz	9.13	0	Good
622	GSM36988.CEL.gz	7.98	0	Good
623	GSM36794.CEL.gz	7.22	0	Good
624	GSM37009.CEL.gz	10.16	0	Good
625	GSM37010.CEL.gz	8.38	0	Good
626	GSM37012.CEL.gz	11.02	0	Good
627	GSM37014.CEL.gz	11.89	0	Good
628	GSM37015.CEL.gz	10.14	0	Good
629	GSM36779.CEL.gz	11.03	0	Good
630	GSM36844.CEL.gz	10.1	0	Good
631	GSM36846.CEL.gz	8.55	0	Good
632	GSM36847.CEL.gz	7.12	0	Good
633	GSM36849.CEL.gz	9.15	0	Good
634	GSM36852.CEL.gz	8.96	0	Good
635	GSM36864.CEL.gz	7.04	0	Good
636	GSM36865.CEL.gz	8.59	0	Good
637	GSM36866.CEL.gz	8.93	0	Good
638	GSM36867.CEL.gz	9	0	Good
639	GSM36890.CEL.gz	9.68	0	Good
640	GSM36912.CEL.gz	8.07	0	Good
641	GSM36914.CEL.gz	6.97	0	Good
642	GSM36915.CEL.gz	8.09	0	Good
643	GSM36932.CEL.gz	7.98	0	Good

Continued on next page

Table S1 – continued from previous page

Count	CEL	t.dmfs	e.dmfs	class label
644	GSM36933.CEL.gz	9.17	0	Good
645	GSM36935.CEL.gz	9.52	0	Good
646	GSM36936.CEL.gz	9.05	0	Good
647	GSM36780.CEL.gz	7.99	0	Good
648	GSM36810.CEL.gz	8.74	0	Good
649	GSM36840.CEL.gz	7.19	0	Good
650	GSM36883.CEL.gz	7.31	0	Good
651	GSM36945.CEL.gz	7.64	0	Good
652	GSM36995.CEL.gz	9.41	0	Good
653	GSM37034.CEL.gz	7.36	0	Good
654	GSM36795.CEL.gz	7.32	0	Good
655	GSM36821.CEL.gz	7.33	0	Good
656	GSM36863.CEL.gz	8.13	0	Good
657	GSM37024.CEL.gz	7.89	0	Good
658	GSM37048.CEL.gz	7.29	0	Good
659	GSM36979.CEL.gz	8.88	0	Good
660	GSM36981.CEL.gz	7.23	0	Good
661	GSM36982.CEL.gz	7.67	0	Good
662	GSM37005.CEL.gz	4.65	1	Poor
663	GSM37007.CEL.gz	4.96	1	Poor
664	GSM37011.CEL.gz	5	1	Poor
665	GSM37016.CEL.gz	9.03	0	Good
666	GSM37032.CEL.gz	7.15	0	Good
667	GSM37033.CEL.gz	13.38	0	Good
668	GSM37043.CEL.gz	13.4	0	Good
669	GSM37044.CEL.gz	9.37	0	Good
670	GSM37045.CEL.gz	10.23	0	Good
671	GSM37046.CEL.gz	7.17	0	Good
672	GSM36887.CEL.gz	8.97	0	Good
673	GSM36889.CEL.gz	9.03	0	Good
674	GSM36892.CEL.gz	9.19	0	Good
675	GSM36894.CEL.gz	9.05	0	Good
676	GSM120649.CEL.gz	11.36	0	Good
677	GSM120653.CEL.gz	8.01	0	Good
678	GSM120651.CEL.gz	7.38	0	Good
679	GSM120654.CEL.gz	8.13	0	Good
680	GSM120655.CEL.gz	0.84	1	Poor
681	GSM120656.CEL.gz	1.88	1	Poor
682	GSM120663.CEL.gz	2.42	1	Poor
683	GSM120664.CEL.gz	6.95	0	Good
684	GSM120652.CEL.gz	1.02	1	Poor
685	GSM120657.CEL.gz	5.1	0	Good
686	GSM120658.CEL.gz	10.24	0	Good
687	GSM120659.CEL.gz	11.67	0	Good
688	GSM120660.CEL.gz	9.72	0	Good
689	GSM120689.CEL.gz	8.13	0	Good
690	GSM120665.CEL.gz	13.07	0	Good

Continued on next page

Table S1 – continued from previous page

Count	CEL	t.dmfs	e.dmfs	class label
691	GSM120666.CEL.gz	7.3	0	Good
692	GSM120667.CEL.gz	5.95	0	Good
693	GSM120671.CEL.gz	1.97	1	Poor
694	GSM120668.CEL.gz	0.6	1	Poor
695	GSM120672.CEL.gz	10.67	0	Good
696	GSM120678.CEL.gz	8.89	0	Good
697	GSM120673.CEL.gz	5.37	0	Good
698	GSM120674.CEL.gz	7.21	0	Good
699	GSM120670.CEL.gz	5.89	0	Good
700	GSM120675.CEL.gz	9.97	0	Good
701	GSM120676.CEL.gz	6.15	0	Good
702	GSM120677.CEL.gz	5.71	0	Good
703	GSM120679.CEL.gz	3.48	1	Poor
704	GSM120687.CEL.gz	0.77	1	Poor
705	GSM120680.CEL.gz	8.62	0	Good
706	GSM120681.CEL.gz	9.61	0	Good
707	GSM120682.CEL.gz	6.37	0	Good
708	GSM120669.CEL.gz	8.04	0	Good
709	GSM120688.CEL.gz	10.86	0	Good
710	GSM120683.CEL.gz	10.59	0	Good
711	GSM120684.CEL.gz	10.95	0	Good
712	GSM120685.CEL.gz	7.34	0	Good
713	GSM120686.CEL.gz	6.75	0	Good
714	GSM120690.CEL.gz	7.33	0	Good
715	GSM120691.CEL.gz	8.58	0	Good
716	GSM120693.CEL.gz	5.23	0	Good
717	GSM120692.CEL.gz	7.4	0	Good
718	GSM120694.CEL.gz	7.19	0	Good
719	GSM120695.CEL.gz	7.5	0	Good
720	GSM120696.CEL.gz	12.65	0	Good
721	GSM120697.CEL.gz	9.31	0	Good
722	GSM120698.CEL.gz	5.13	0	Good
723	GSM120699.CEL.gz	6.66	0	Good
724	GSM120700.CEL.gz	6.07	0	Good
725	GSM120701.CEL.gz	5.76	0	Good
726	GSM120702.CEL.gz	0.36	1	Poor
727	GSM120703.CEL.gz	7.62	0	Good
728	GSM120704.CEL.gz	7.61	0	Good
729	GSM120707.CEL.gz	1.8	1	Poor
730	GSM120705.CEL.gz	6.92	0	Good
731	GSM120706.CEL.gz	8.53	0	Good
732	GSM282374.CEL.gz	5.92	0	Good
733	GSM282375.CEL.gz	4.83	1	Poor
734	GSM282376.CEL.gz	5.67	0	Good
735	GSM282377.CEL.gz	8.58	0	Good
736	GSM282378.CEL.gz	7.75	0	Good
737	GSM282379.CEL.gz	9.25	0	Good

Continued on next page

Table S1 – continued from previous page

Count	CEL	t.dmfs	e.dmfs	class label
738	GSM282380.CEL.gz	7.08	0	Good
739	GSM282381.CEL.gz	8.17	0	Good
740	GSM282382.CEL.gz	6.75	0	Good
741	GSM282383.CEL.gz	6.58	0	Good
742	GSM282384.CEL.gz	6.67	0	Good
743	GSM282385.CEL.gz	7.42	0	Good
744	GSM282386.CEL.gz	7	0	Good
745	GSM282388.CEL.gz	7.33	0	Good
746	GSM282389.CEL.gz	6.67	0	Good
747	GSM282390.CEL.gz	5.75	0	Good
748	GSM282391.CEL.gz	7.75	0	Good
749	GSM282392.CEL.gz	7.33	0	Good
750	GSM282393.CEL.gz	8	0	Good
751	GSM282395.CEL.gz	6.58	0	Good
752	GSM282396.CEL.gz	2.17	1	Poor
753	GSM282397.CEL.gz	6.83	0	Good
754	GSM282398.CEL.gz	1.25	1	Poor
755	GSM282399.CEL.gz	6	0	Good
756	GSM282400.CEL.gz	7.42	0	Good
757	GSM282401.CEL.gz	6.5	0	Good
758	GSM282402.CEL.gz	5.75	0	Good
759	GSM282404.CEL.gz	6.08	0	Good
760	GSM282405.CEL.gz	5.33	0	Good
761	GSM282406.CEL.gz	7.08	0	Good
762	GSM282407.CEL.gz	5.92	0	Good
763	GSM282409.CEL.gz	7.92	0	Good
764	GSM282410.CEL.gz	3.08	1	Poor
765	GSM282411.CEL.gz	7.92	0	Good
766	GSM282413.CEL.gz	1.42	1	Poor
767	GSM282414.CEL.gz	5.75	0	Good
768	GSM282415.CEL.gz	6.58	0	Good
769	GSM282416.CEL.gz	7.58	0	Good
770	GSM282417.CEL.gz	6	0	Good
771	GSM282418.CEL.gz	2.92	1	Poor
772	GSM282419.CEL.gz	6.17	0	Good
773	GSM282420.CEL.gz	10.92	0	Good
774	GSM282421.CEL.gz	9.92	0	Good
775	GSM282422.CEL.gz	10.92	0	Good
776	GSM282423.CEL.gz	5.5	0	Good
777	GSM282424.CEL.gz	7.58	0	Good
778	GSM282425.CEL.gz	8.17	0	Good
779	GSM282426.CEL.gz	3.33	1	Poor
780	GSM282427.CEL.gz	10	0	Good
781	GSM282428.CEL.gz	6.58	0	Good
782	GSM282429.CEL.gz	10.17	0	Good
783	GSM282430.CEL.gz	9.92	0	Good
784	GSM282431.CEL.gz	3.67	1	Poor

Continued on next page

Table S1 – continued from previous page

Count	CEL	t.dmfs	e.dmfs	class label
785	GSM282432.CEL.gz	9.42	0	Good
786	GSM282433.CEL.gz	9.75	0	Good
787	GSM282434.CEL.gz	5.5	0	Good
788	GSM282435.CEL.gz	9.58	0	Good
789	GSM282436.CEL.gz	5.33	0	Good
790	GSM282437.CEL.gz	9.83	0	Good
791	GSM282438.CEL.gz	7.75	0	Good
792	GSM282439.CEL.gz	8.25	0	Good
793	GSM282440.CEL.gz	8.83	0	Good
794	GSM282441.CEL.gz	7.17	0	Good
795	GSM282443.CEL.gz	10.17	0	Good
796	GSM282444.CEL.gz	10.67	0	Good
797	GSM282445.CEL.gz	10.33	0	Good
798	GSM282446.CEL.gz	9.5	0	Good
799	GSM282447.CEL.gz	9.42	0	Good
800	GSM282448.CEL.gz	10.17	0	Good
801	GSM282449.CEL.gz	10.33	0	Good
802	GSM282450.CEL.gz	9.58	0	Good
803	GSM282452.CEL.gz	5.83	0	Good
804	GSM282453.CEL.gz	11.75	0	Good
805	GSM282454.CEL.gz	0.67	1	Poor
806	GSM282455.CEL.gz	12.17	0	Good
807	GSM282456.CEL.gz	10.92	0	Good
808	GSM282457.CEL.gz	12.33	0	Good
809	GSM282458.CEL.gz	11.08	0	Good
810	GSM282459.CEL.gz	13.25	0	Good
811	GSM282461.CEL.gz	2.5	1	Poor
812	GSM282462.CEL.gz	15.42	0	Good
813	GSM282464.CEL.gz	16.58	0	Good
814	GSM282465.CEL.gz	7.5	0	Good
815	GSM282466.CEL.gz	15.08	0	Good
816	GSM282467.CEL.gz	15.83	0	Good
817	GSM282469.CEL.gz	12.67	0	Good
818	GSM282470.CEL.gz	1.42	1	Poor
819	GSM282471.CEL.gz	1.92	1	Poor
820	GSM282472.CEL.gz	12.58	0	Good
821	GSM282473.CEL.gz	13.33	0	Good
822	GSM282474.CEL.gz	11.92	0	Good
823	GSM282475.CEL.gz	12.17	0	Good
824	GSM282476.CEL.gz	10.83	0	Good
825	GSM282477.CEL.gz	11.58	0	Good
826	GSM282478.CEL.gz	1.25	1	Poor
827	GSM282480.CEL.gz	16.75	0	Good
828	GSM282481.CEL.gz	13.17	0	Good
829	GSM282486.CEL.gz	14.17	0	Good
830	GSM282487.CEL.gz	17.08	0	Good
831	GSM282489.CEL.gz	13.33	0	Good

Continued on next page

Table S1 – continued from previous page

Count	CEL	t.dmfs	e.dmfs	class label
832	GSM282490.CEL.gz	5.83	0	Good
833	GSM282491.CEL.gz	2.5	1	Poor
834	GSM282493.CEL.gz	15.92	0	Good
835	GSM282494.CEL.gz	15.92	0	Good
836	GSM282495.CEL.gz	20	0	Good
837	GSM282496.CEL.gz	5.42	0	Good
838	GSM282497.CEL.gz	7.75	0	Good
839	GSM282498.CEL.gz	16.08	0	Good
840	GSM282499.CEL.gz	15.75	0	Good
841	GSM282500.CEL.gz	16	0	Good
842	GSM282502.CEL.gz	16.92	0	Good
843	GSM282503.CEL.gz	15	0	Good
844	GSM282504.CEL.gz	13.58	0	Good
845	GSM282505.CEL.gz	14.75	0	Good
846	GSM282508.CEL.gz	10.83	0	Good
847	GSM282509.CEL.gz	6.58	0	Good
848	GSM282510.CEL.gz	0.58	1	Poor
849	GSM282511.CEL.gz	11.17	0	Good
850	GSM282513.CEL.gz	8.58	0	Good
851	GSM282514.CEL.gz	13.83	0	Good
852	GSM282516.CEL.gz	8.42	0	Good
853	GSM282518.CEL.gz	10.67	0	Good
854	GSM282519.CEL.gz	11	0	Good
855	GSM282520.CEL.gz	1.83	1	Poor
856	GSM282521.CEL.gz	12.5	0	Good
857	GSM282522.CEL.gz	3.75	1	Poor
858	GSM282523.CEL.gz	12.92	0	Good
859	GSM282524.CEL.gz	11.92	0	Good
860	GSM282525.CEL.gz	0.5	1	Poor
861	GSM282526.CEL.gz	6.42	0	Good
862	GSM282527.CEL.gz	6.83	0	Good
863	GSM282528.CEL.gz	11.08	0	Good
864	GSM282529.CEL.gz	7.25	0	Good
865	GSM282531.CEL.gz	12.33	0	Good
866	GSM282532.CEL.gz	6.25	0	Good
867	GSM282534.CEL.gz	10.42	0	Good
868	GSM282535.CEL.gz	3.08	1	Poor
869	GSM282536.CEL.gz	16.5	0	Good
870	GSM282537.CEL.gz	11.08	0	Good
871	GSM282538.CEL.gz	1.5	1	Poor
872	GSM282539.CEL.gz	9.75	0	Good
873	GSM282540.CEL.gz	7.83	0	Good
874	GSM282541.CEL.gz	8.08	0	Good
875	GSM282542.CEL.gz	10.5	0	Good
876	GSM282543.CEL.gz	6	0	Good
877	GSM282544.CEL.gz	4.67	1	Poor
878	GSM282545.CEL.gz	11.75	0	Good

Continued on next page

Table S1 – continued from previous page

Count	CEL	t.dmfs	e.dmfs	class label
879	GSM282546.CEL.gz	6	0	Good
880	GSM282547.CEL.gz	9.33	0	Good
881	GSM282548.CEL.gz	7.75	0	Good
882	GSM282550.CEL.gz	7.58	0	Good
883	GSM282551.CEL.gz	0.67	1	Poor
884	GSM282558.CEL.gz	5.42	0	Good
885	GSM282559.CEL.gz	5.08	0	Good
886	GSM282560.CEL.gz	5.42	0	Good
887	GSM282564.CEL.gz	6.33	0	Good
888	GSM282566.CEL.gz	3.5	1	Poor
889	GSM282567.CEL.gz	1.17	1	Poor
890	GSM282568.CEL.gz	3.83	1	Poor
891	GSM282570.CEL.gz	2.67	1	Poor
892	GSM282571.CEL.gz	5.17	0	Good

Table S1. Overview event prediction compendium
