# Supplementary material for: An Evaluation Protocol for Subtype-Specific Breast Cancer Event Prediction
Source: PLoS One. 2011 Jul 8;6(7):e21681. doi: 10.1371/journal.pone.0021681 (PMC3132736; doi:10.1371/journal.pone.0021681)
Supplement: Supporting Information S2 — Additional classification results in which the ranking strategy, the predictor, and cross-validation scheme, respectively, have been altered compared to the setup corresponding to Figure 5 . The ranking strategy was altered from a ranking by moderated- statistics to a ranking by signal-to-noise-ratio statistics (SNR). In addition, the nearest centroid (NC) predictor was replaced by the random forest (RF) predictor [40], which is a highly non-linear predictor. Finally, the = 10-fold cross-validation strategy was changed to 3-fold cross-validation, 5-fold cross-validation, and leave-one-out cross-validation (LOOCV), respectively. (PDF) [file pone.0021681.s002.pdf]

Supporting Information S2 - An Evaluation Protocol for Subtype-Specific Breast Cancer Event Prediction

Herman MJ Sontrop[1], Wim FJ Verhaegh[1], Marcel JT Reinders[2,4], Perry D Moerland[3,4,*]
1 Molecular Diagnostics Department, Philips Research, High Tech Campus 11, 5656 AE Eindhoven, The Netherlands
2 Delft Bioinformatics Lab, Delft University of Technology, Mekelweg 4, 2628 CD Delft, The Netherlands
3 Bioinformatics Laboratory, Department of Clinical Epidemiology, Biostatistics, and Bioinformatics, Academic Medical Center, Meibergdreef 9, 1105 AZ Amsterdam, The Netherlands
4 Netherlands Bioinformatics Centre, Geert Grooteplein 28, 6525 GA Nijmegen, The Netherlands
∗ E-mail: p.d.moerland@amc.uva.nl

Additional classification results

Figures S1-S5 show additional classification results in which the ranking strategy, the predictor, and cross-validation scheme, respectively, have been altered compared to the setup corresponding to Figure 5 of the main text. In Figure S1 the ranking strategy was altered from a ranking by moderated-t statistics to a ranking by signal-to-noise-ratio statistics (SNR). For a definition of SNR see [1]. Figure S2 presents the results when replacing the nearest centroid (NC) predictor, a linear classifier, by the random forest (RF) predictor [2], which is a highly non-linear predictor. The RF predictors were fit using the R package *randomForests*, with parameters $\mathsf{ntree} = 1000$, $\mathsf{mty} = \sqrt{200}$, $\mathsf{nodesize} = 1$. As training is computationally demanding for this predictor a fixed signature size of 200 was used. Finally, the strata and sampsize parameters were set such that each tree was built using an equal number of positive and negative training samples in order to prevent an overfit towards the majority class.
Figures S3-S5 present the results when the $K_{out} = 10$-fold cross-validation strategy was changed to 3-fold cross-validation, 5-fold cross-validation, and leave-one-out cross-validation (LOOCV), respectively. Note that LOOCV is not a special case of our protocol as the protocol is based on stratified cross-validation, in which for each stratum one or more samples are left out and not one from the entire dataset, as is the case in LOOCV. In the LOOCV experiments each sample f is, therefore, left out individually, with $V_f = \{f\}$ and $T_f = D - \{f\}$. The set $T^{\mathrm{tp}}_{s,f}$ consists of all training samples of subtype s. The set $T^{\mathrm{un}}_{s,f}$ is constructed by randomly sampling the same number of positive and negative samples from T_f as present in $T^{\mathrm{tp}}_{s,f}$.

References

1. Sontrop H, Moerland P, Van Den Ham R, Reinders M, Verhaegh W (2009) A comprehensive sensitivity analysis of microarray breast cancer classification under feature variability. BMC Bioinformatics 10: 389.

2. Breiman L (2001) Random forests. Machine Learning 45: 5–32.

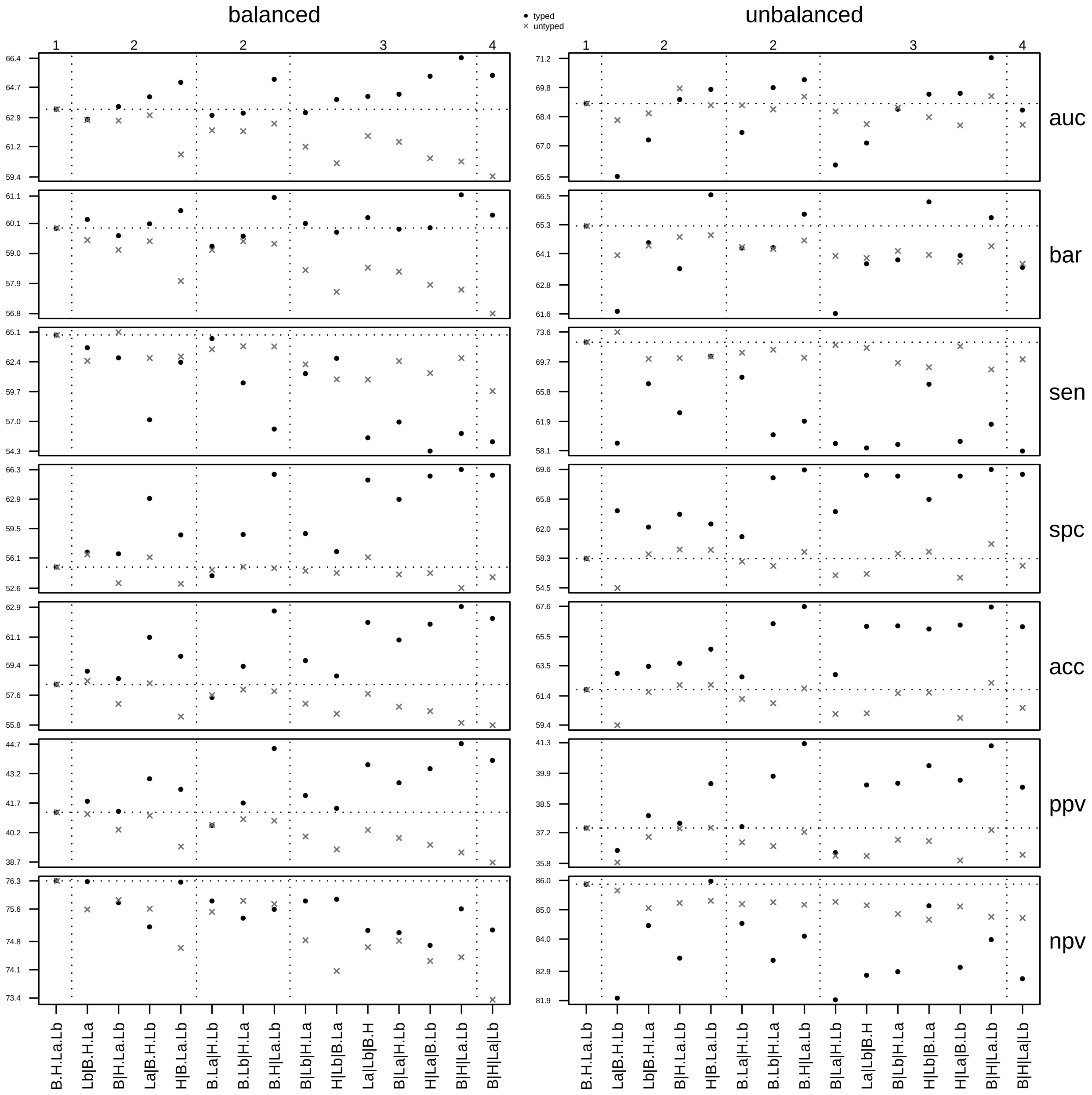

Figure S1. NC with signal-to-noise-ratio based ranking. Performance overview of overall performance corresponding to the 15 distinct partitioning schemes w.r.t. the elementary subtype set $S_e = \{L_a, L_b, H, B\}$, that represents the subtypes lumA, lumB, Her2 and basal, respectively. The left panel corresponds to experiments involving the balanced compendia B, while the right panel corresponds to experiments involving the full unbalanced compendium D. In each panel the top numbers $\{1, 2, 3, 4\}$ indicate the number of different parts in each of the partitions, while the bottom line identifies the precise makeup of the various partitions e.g. the notation B|H|La.Lb indicates a partition into three parts, involving separate basal and Her2 groups, while having a combined luminal group. In each panel the coarsest partition is situated at the outer left, which corresponds to the baseline predictor, that is, a single predictor that targets all samples. The most refined partition is situated at the outer right, which uses a separate predictor for each elementary subtype. A horizontal dotted line indicates the performance of the baseline predictors. Vertical dotted lines are used to group the partitions by their number of parts, as indicated by the top numbers. Results represent averages over 100 repeats. Rows represent seven frequently used performance indicators: area under curve (auc), balanced accuracy (bar), sensitivity (sen), specificity (spc), accuracy (acc), positive predictive value (ppv) and negative predictive value (npv). Performance for typed predictors is indicated with a dot, performance for untyped predictors with a cross.

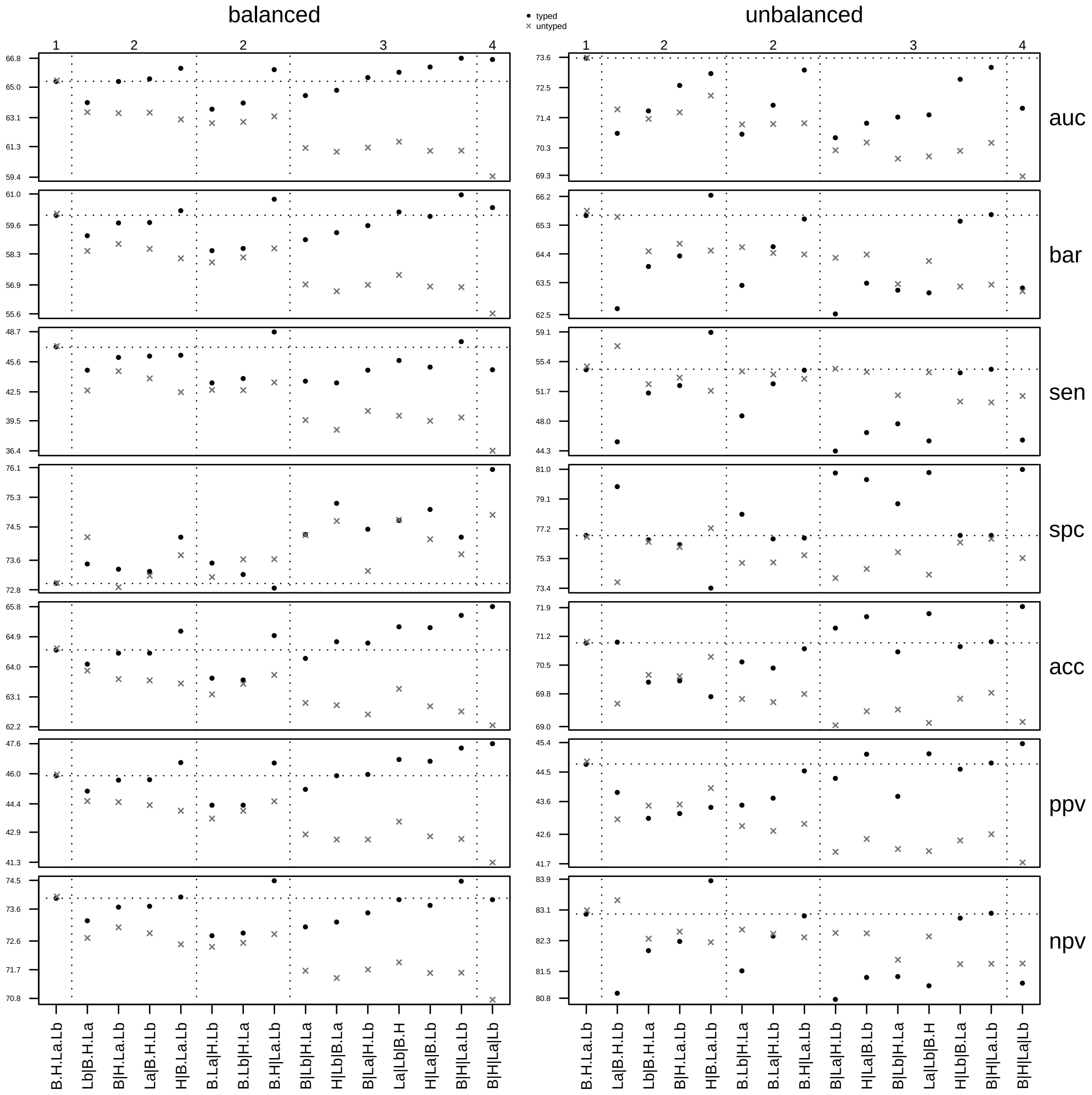

Figure S2. Random Forest. Performance overview of overall performance corresponding to the 15 distinct partitioning schemes w.r.t. the elementary subtype set $S_e = \{L_a, L_b, H, B\}$, that represents the subtypes lumA, lumB, Her2 and basal, respectively. The left panel corresponds to experiments involving the balanced compendia B, while the right panel corresponds to experiments involving the full unbalanced compendium D. In each panel the top numbers $\{1, 2, 3, 4\}$ indicate the number of different parts in each of the partitions, while the bottom line identifies the precise makeup of the various partitions e.g. the notation B|H|La.Lb indicates a partition into three parts, involving separate basal and Her2 groups, while having a combined luminal group. In each panel the coarsest partition is situated at the outer left, which corresponds to the baseline predictor, that is, a single predictor that targets all samples. The most refined partition is situated at the outer right, which uses a separate predictor for each elementary subtype. A horizontal dotted line indicates the performance of the baseline predictors. Vertical dotted lines are used to group the partitions by their number of parts, as indicated by the top numbers. Results represent averages over 100 repeats. Rows represent seven frequently used performance indicators: area under curve (auc), balanced accuracy (bar), sensitivity (sen), specificity (spc), accuracy (acc), positive predictive value (ppv) and negative predictive value (npv). Performance for typed predictors is indicated with a dot, performance for untyped predictors with a cross.

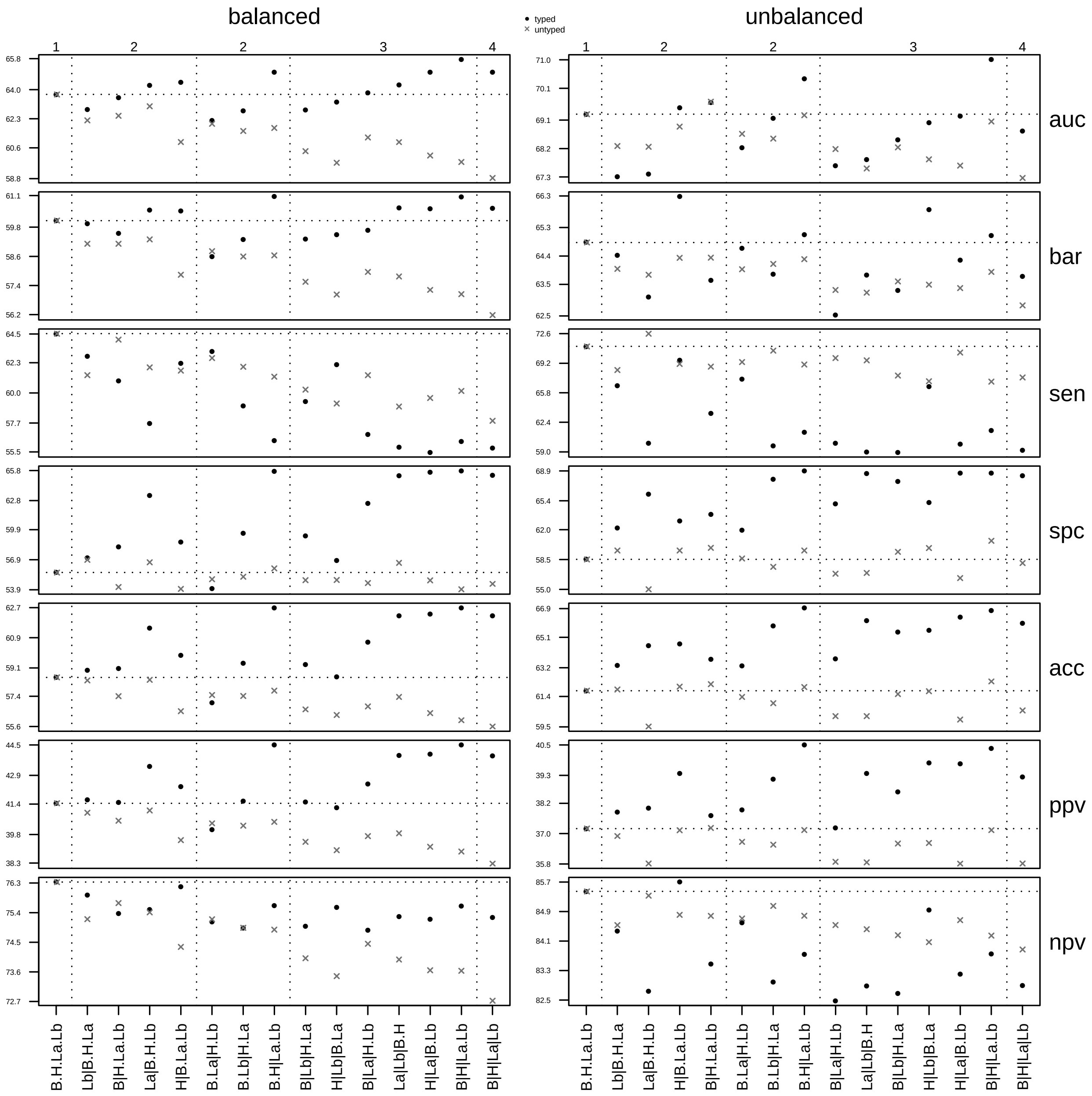

Figure S3. 3-fold cross-validation NC. Performance overview of overall performance corresponding to the 15 distinct partitioning schemes w.r.t. the elementary subtype set $S_e = \{L_a, L_b, H, B\}$, that represents the subtypes lumA, lumB, Her2 and basal, respectively. The left panel corresponds to experiments involving the balanced compendia B, while the right panel corresponds to experiments involving the full unbalanced compendium D. In each panel the top numbers $\{1, 2, 3, 4\}$ indicate the number of different parts in each of the partitions, while the bottom line identifies the precise makeup of the various partitions e.g. the notation B|H|La.Lb indicates a partition into three parts, involving separate basal and Her2 groups, while having a combined luminal group. In each panel the coarsest partition is situated at the outer left, which corresponds to the baseline predictor, that is, a single predictor that targets all samples. The most refined partition is situated at the outer right, which uses a separate predictor for each elementary subtype. A horizontal dotted line indicates the performance of the baseline predictors. Vertical dotted lines are used to group the partitions by their number of parts, as indicated by the top numbers. Results represent averages over 100 repeats. Rows represent seven frequently used performance indicators: area under curve (auc), balanced accuracy (bar), sensitivity (sen), specificity (spc), accuracy (acc), positive predictive value (ppv) and negative predictive value (npv). Performance for typed predictors is indicated with a dot, performance for untyped predictors with a cross.

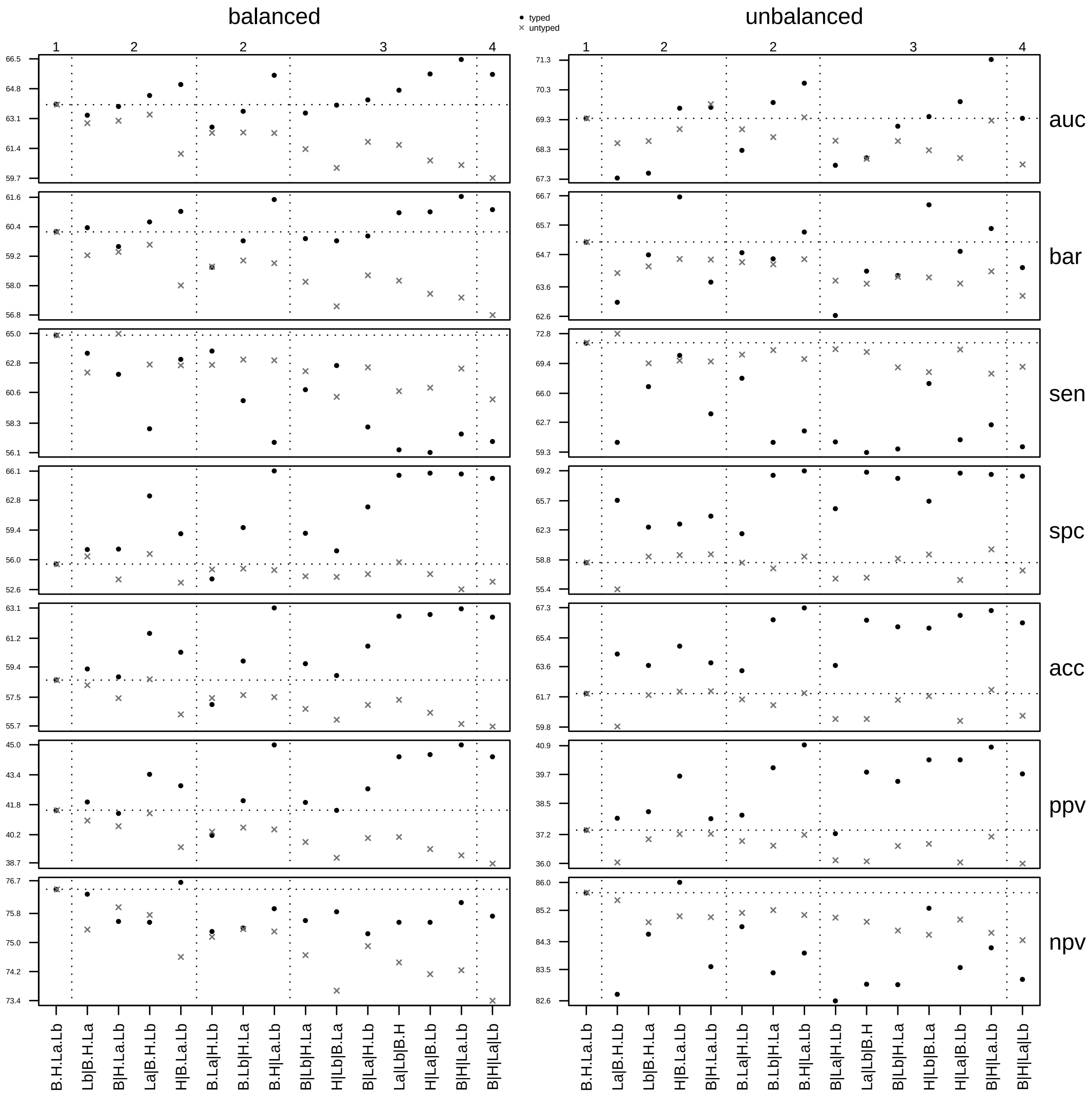

Figure S4. 5-fold cross-validation NC. Performance overview of overall performance corresponding to the 15 distinct partitioning schemes w.r.t. the elementary subtype set $S_e = \{L_a, L_b, H, B\}$, that represents the subtypes lumA, lumB, Her2 and basal, respectively. The left panel corresponds to experiments involving the balanced compendia B, while the right panel corresponds to experiments involving the full unbalanced compendium D. In each panel the top numbers $\{1, 2, 3, 4\}$ indicate the number of different parts in each of the partitions, while the bottom line identifies the precise makeup of the various partitions e.g. the notation B|H|La.Lb indicates a partition into three parts, involving separate basal and Her2 groups, while having a combined luminal group. In each panel the coarsest partition is situated at the outer left, which corresponds to the baseline predictor, that is, a single predictor that targets all samples. The most refined partition is situated at the outer right, which uses a separate predictor for each elementary subtype. A horizontal dotted line indicates the performance of the baseline predictors. Vertical dotted lines are used to group the partitions by their number of parts, as indicated by the top numbers. Results represent averages over 100 repeats. Rows represent seven frequently used performance indicators: area under curve (auc), balanced accuracy (bar), sensitivity (sen), specificity (spc), accuracy (acc), positive predictive value (ppv) and negative predictive value (npv). Performance for typed predictors is indicated with a dot, performance for untyped predictors with a cross.

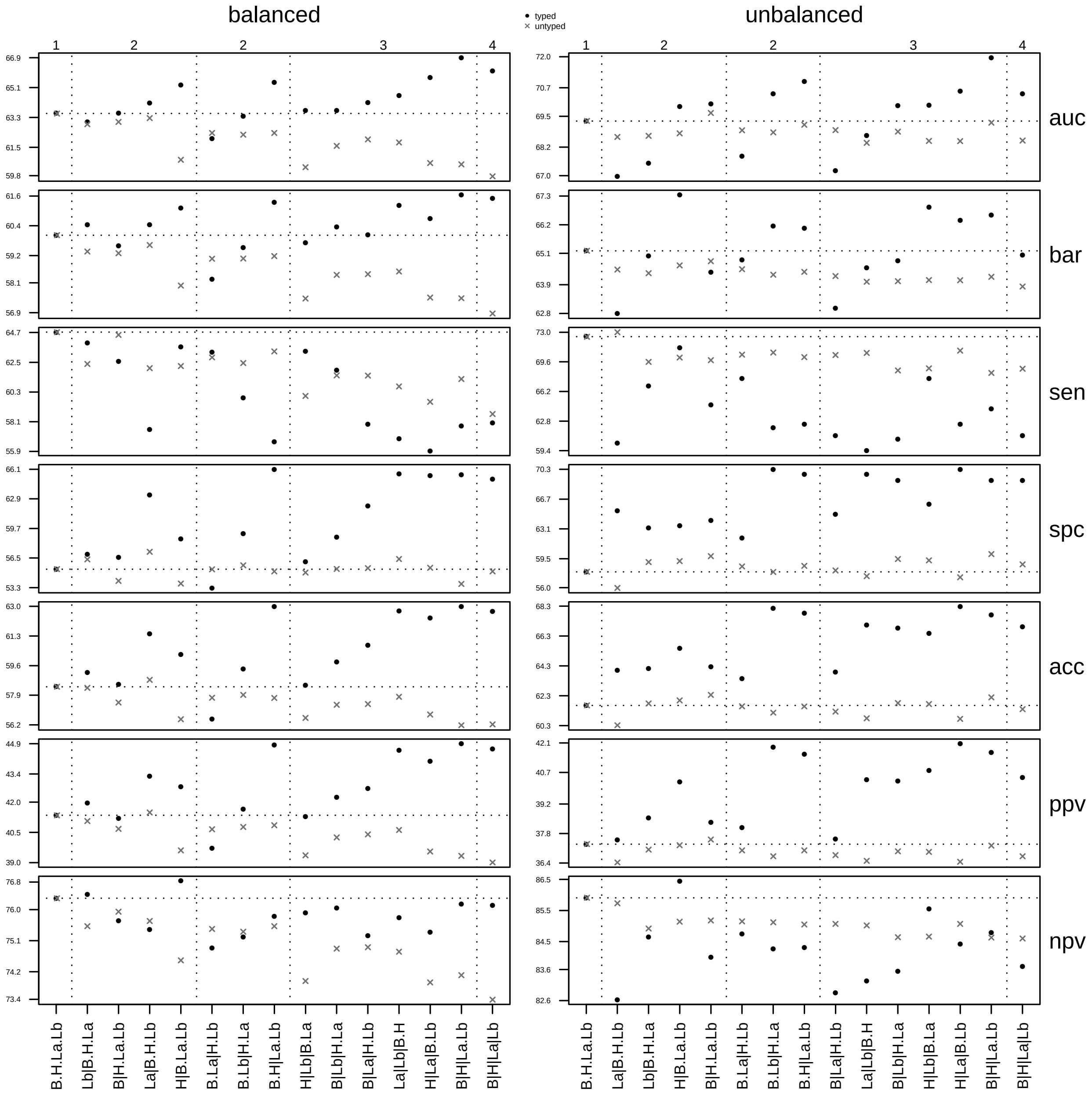

Figure S5. Leave-one-out cross-validation NC. Performance overview of overall performance corresponding to the 15 distinct partitioning schemes w.r.t. the elementary subtype set $S_e = \{L_a, L_b, H, B\}$, that represents the subtypes lumA, lumB, Her2 and basal, respectively. The left panel corresponds to experiments involving the balanced compendia B, while the right panel corresponds to experiments involving the full unbalanced compendium D. In each panel the top numbers $\{1, 2, 3, 4\}$ indicate the number of different parts in each of the partitions, while the bottom line identifies the precise makeup of the various partitions e.g. the notation B|H|La.Lb indicates a partition into three parts, involving separate basal and Her2 groups, while having a combined luminal group. In each panel the coarsest partition is situated at the outer left, which corresponds to the baseline predictor, that is, a single predictor that targets all samples. The most refined partition is situated at the outer right, which uses a separate predictor for each elementary subtype. A horizontal dotted line indicates the performance of the baseline predictors. Vertical dotted lines are used to group the partitions by their number of parts, as indicated by the top numbers. Results represent averages over 100 repeats. Rows represent seven frequently used performance indicators: area under curve (auc), balanced accuracy (bar), sensitivity (sen), specificity (spc), accuracy (acc), positive predictive value (ppv) and negative predictive value (npv). Performance for typed predictors is indicated with a dot, performance for untyped predictors with a cross.
