# Supplementary material for: An Evaluation Protocol for Subtype-Specific Breast Cancer Event Prediction
Source: PLoS One. 2011 Jul 8;6(7):e21681. doi: 10.1371/journal.pone.0021681 (PMC3132736; doi:10.1371/journal.pone.0021681)
Supplement: Supporting Information S3 — Complete set of performances tables (similar to Tables 2 and 3 of the main text) corresponding to Figure 5 . Each table provides a performance overview per elementary subtype: typed (tp) versus untyped (un) predictors, for a given partition, which is stated in the caption. The highest value for a paired typed and untyped performance measure is set in italic. If the difference is significant (two sided paired -test, ) the entry is set in bold. (PDF) [file pone.0021681.s003.pdf]

Supporting Information S3: An Evaluation Protocol for Subtype-Specific Breast Cancer Event Prediction

Herman MJ Sontrop[1], Wim FJ Verhaegh[1], Marcel JT Reinders[2,4], Perry D Moerland[3,4,*]

1 Molecular Diagnostics Department, Philips Research, High Tech Campus 11, 5656 AE Eindhoven, The Netherlands

2 Delft Bioinformatics Lab, Delft University of Technology, Mekelweg 4, 2628 CD Delft, The Netherlands

3 Bioinformatics Laboratory, Department of Clinical Epidemiology, Biostatistics, and Bioinformatics, Academic Medical Center, Meibergdreef 9, 1105 AZ Amsterdam, The Netherlands

4 Netherlands Bioinformatics Centre, Geert Grooteplein 28, 6525 GA Nijmegen, The Netherlands

∗ E-mail: p.d.moerland@amc.uva.nl

Performance tables Nearest Centroid on balanced compendia

Table S2. Typed (tp) vs. untyped (un) predictor performance.

		lumA	lumB	basal	Her2	overall
	auc	57.1	64.8	61.2	75.3	64.1
	bar	54.9	58.5	57.4	70.0	60.2
	sen	64.8	76.2	43.9	74.3	64.8
tp	spc	45.1	40.7	70.8	65.6	55.5
	acc	51.5	52.3	62.0	68.4	58.6
	ppv	36.6	38.6	42.4	51.3	41.5
	npv	72.3	78.0	72.2	84.0	76.5
	auc	57.1	64.8	61.2	75.3	64.1
	bar	54.9	58.5	57.4	70.0	60.2
	sen	64.8	76.2	43.9	74.3	64.8
up	spc	45.1	40.7	70.8	65.6	55.5
	acc	51.5	52.3	62.0	68.4	58.6
	ppv	36.6	38.6	42.4	51.3	41.5
	npv	72.3	78.0	72.2	84.0	76.5

B.H.La.Lb

Table S3. Typed (tp) vs. untyped (un) predictor performance.

		lumA	lumB	basal	Her2	overall
tp	auc	***59.4***	***65.9***	***60.6***	72.2	***64.0***
	bar	***56.4***	58.5	***56.7***	67.5	*59.8*
	sen	53.2	***78.1***	44.6	*73.9*	62.5
	spc	***59.7***	39.0	***68.8***	61.0	***57.1***
	acc	***57.5***	51.8	***60.9***	65.2	***58.9***
	ppv	***39.3***	38.4	***40.9***	48.2	***41.5***
	npv	*72.4*	*78.7*	*72.0*	82.7	75.8
up	auc	57.0	64.4	57.1	***73.6***	63.0
	bar	55.0	*58.9*	54.7	***68.8***	59.3
	sen	***63.1***	75.4	*48.1*	73.6	***65.1***
	spc	46.9	***42.3***	61.3	***63.9***	53.6
	acc	52.2	***53.1***	57.0	***67.1***	57.3
	ppv	36.7	*39.0*	37.9	***49.9***	40.6
	npv	72.2	78.1	70.9	*83.3*	*75.9*

$B|H.La.Lb$

Table S4. Typed (tp) vs. untyped (un) predictor performance.

		lumA	lumB	basal	Her2	overall
tp	auc	***59.1***	***66.4***	59.3	***74.7***	***65.7***
	bar	***57.2***	***61.2***	55.2	***71.5***	***61.3***
	sen	59.0	72.4	*46.3*	***75.9***	*63.4*
	spc	***55.5***	***50.0***	64.2	***67.2***	***59.2***
	acc	***56.6***	***57.4***	58.3	***70.0***	***60.6***
	ppv	***39.7***	***41.6***	38.9	***52.9***	***43.1***
	npv	***73.5***	*79.1*	71.3	***85.1***	***76.9***
up	auc	57.0	64.5	***60.8***	65.9	61.2
	bar	55.1	59.1	***57.3***	61.7	58.3
	sen	***63.3***	***75.6***	45.2	67.0	62.8
	spc	46.8	42.6	***69.5***	56.5	53.8
	acc	52.2	53.4	***61.6***	59.9	56.8
	ppv	36.8	39.1	***42.1***	43.1	39.8
	npv	72.3	78.3	***72.3***	77.9	74.8

$H|B.La.Lb$

Table S5. Typed (tp) vs. untyped (un) predictor performance.

		lumA	lumB	basal	Her2	overall
tp	auc	***61.5***	63.2	***61.6***	***75.6***	***64.6***
	bar	***56.3***	57.5	*57.4*	***70.8***	***60.5***
	sen	37.5	*74.9*	43.8	*73.9*	57.5
	spc	***75.1***	40.1	***70.9***	***67.7***	***63.4***
	acc	***62.8***	51.5	***62.0***	***69.7***	***61.5***
	ppv	***42.4***	37.9	*42.3*	***52.7***	***43.4***
	npv	*71.2*	76.8	*72.2*	***84.2***	75.4
up	auc	55.3	*63.9*	60.4	73.6	63.1
	bar	53.8	***58.5***	57.1	68.5	59.5
	sen	***56.3***	74.2	*45.3*	73.3	***62.3***
	spc	51.3	***42.8***	69.0	63.7	56.7
	acc	52.9	***53.1***	61.2	66.8	58.5
	ppv	36.1	***38.7***	41.7	49.7	41.2
	npv	70.7	*77.5*	72.2	83.1	*75.6*

$La|B.H.Lb$

Table S6. Typed (tp) vs. untyped (un) predictor performance.

		lumA	lumB	basal	Her2	overall
tp	auc	55.5	***65.0***	60.4	*74.3*	*63.1*
	bar	54.2	***60.8***	*57.0*	*69.5*	***60.4***
	sen	***66.1***	***71.7***	43.7	73.4	***63.7***
	spc	42.4	*49.8*	*70.2*	***65.6***	*57.0*
	acc	50.1	***57.0***	*61.6*	***68.2***	***59.2***
	ppv	35.9	***41.2***	*41.7*	*51.0*	***41.9***
	npv	71.8	***78.4***	*72.0*	83.5	***76.4***
up	auc	***57.0***	60.6	*60.8*	74.1	63.0
	bar	*55.0*	57.0	56.8	69.2	59.5
	sen	63.2	66.4	*44.3*	***74.6***	62.1
	spc	***46.7***	47.5	69.4	63.8	56.8
	acc	***52.1***	53.7	61.2	67.3	58.6
	ppv	*36.7*	38.1	41.3	50.2	41.2
	npv	*72.2*	74.7	71.9	*83.8*	75.5

$Lb|B.H.La$

Table S7. Typed (tp) vs. untyped (un) predictor performance.

		lumA	lumB	basal	Her2	overall
	auc	***65.6***	***68.2***	*60.5*	***74.4***	***65.7***
	bar	***57.6***	***61.7***	*57.1*	***69.9***	***61.6***
	sen	32.1	***78.9***	44.3	*72.6*	57.0
tp	spc	***83.2***	44.5	***69.8***	***67.1***	***66.1***
	acc	***66.5***	***55.7***	***61.5***	***68.9***	***63.1***
	ppv	***48.6***	***41.0***	*41.7*	***51.8***	***45.0***
	npv	71.7	***81.5***	72.0	***83.4***	*76.0*
	auc	56.5	63.9	59.8	71.4	62.7
	bar	55.4	59.0	56.8	66.4	59.4
	sen	***61.3***	72.9	***47.7***	72.2	***63.5***
up	spc	49.4	*45.2*	65.9	60.6	55.3
	acc	53.3	54.2	59.9	64.4	58.0
	ppv	37.2	39.4	40.6	47.3	40.9
	npv	*72.4*	77.6	*72.2*	81.8	75.7

$B.H|La.Lb$

Table S8. Typed (tp) vs. untyped (un) predictor performance.

		lumA	lumB	basal	Her2	overall
	auc	55.7	62.5	57.0	***74.1***	***62.9***
	bar	54.9	57.1	54.0	***70.6***	*59.1*
	sen	*63.9*	***76.3***	42.6	***73.8***	*64.2*
tp	spc	45.8	37.8	65.3	***67.3***	54.1
	acc	51.7	50.4	57.9	***69.4***	57.4
	ppv	36.7	37.4	37.3	***52.4***	40.5
	npv	72.2	76.8	70.2	***84.1***	*75.7*
	auc	*56.7*	*62.8*	***59.8***	70.5	62.2
	bar	*55.3*	***58.5***	***56.2***	65.9	59.0
	sen	61.4	72.0	***46.7***	71.1	62.8
up	spc	***49.2***	***45.1***	*65.6*	60.7	***55.2***
	acc	***53.2***	***53.9***	***59.4***	64.1	*57.7*
	ppv	*37.2*	***39.0***	***40.0***	47.0	*40.5*
	npv	*72.5*	*76.9*	***71.8***	81.3	75.3

$B.La|H.Lb$

Table S9. Typed (tp) vs. untyped (un) predictor performance.

		lumA	lumB	basal	Her2	overall
tp	auc	*56.8*	*64.0*	***61.6***	*72.2*	***63.7***
	bar	*54.8*	*59.4*	56.3	***68.7***	*59.8*
	sen	55.6	67.7	43.3	***74.8***	60.3
	spc	***53.9***	***51.2***	***69.4***	***62.7***	***59.3***
	acc	***54.5***	***56.6***	*60.8*	***66.6***	***59.6***
	ppv	*37.1*	***40.5***	*40.9*	***49.5***	***41.9***
	npv	71.4	76.8	71.6	***83.6***	75.4
up	auc	56.4	63.2	59.5	71.2	62.3
	bar	54.8	59.0	*56.9*	66.6	59.3
	sen	***60.1***	***72.7***	***47.8***	72.2	***63.2***
	spc	49.4	45.3	66.0	61.0	55.4
	acc	52.9	54.3	60.1	64.7	58.0
	ppv	36.8	39.4	40.8	47.6	40.9
	npv	*71.8*	*77.6*	*72.3*	81.9	*75.6*

$B.Lb|H.La$

Table S10. Typed (tp) vs. untyped (un) predictor performance.

		lumA	lumB	basal	Her2	overall
tp	auc	***65.6***	***68.2***	***60.6***	***74.7***	***66.9***
	bar	***57.6***	***61.7***	***56.7***	***71.5***	***61.9***
	sen	32.1	***78.9***	44.6	***75.9***	57.9
	spc	***83.2***	44.5	***68.8***	***67.2***	***65.9***
	acc	***66.5***	***55.7***	***60.9***	***70.0***	***63.3***
	ppv	***48.6***	***41.0***	***40.9***	***52.9***	***45.2***
	npv	71.7	***81.5***	*72.0*	***85.1***	***76.3***
up	auc	56.5	63.9	57.1	65.9	60.5
	bar	55.4	59.0	54.7	61.7	57.7
	sen	***61.3***	72.9	*48.1*	67.0	***62.3***
	spc	49.4	*45.2*	61.3	56.5	53.1
	acc	53.3	54.2	57.0	59.9	56.1
	ppv	37.2	39.4	37.9	43.1	39.3
	npv	*72.4*	77.6	70.9	77.9	74.4

$B|H|La.Lb$

Table S11. Typed (tp) vs. untyped (un) predictor performance.

		lumA	lumB	basal	Her2	overall
	auc	***61.5***	62.5	***60.6***	***74.1***	***64.4***
	bar	***56.3***	57.1	***56.7***	***70.6***	***60.2***
	sen	37.5	***76.3***	44.6	***73.8***	58.1
tp	spc	***75.1***	37.8	***68.8***	***67.3***	***62.3***
	acc	***62.8***	50.4	***60.9***	***69.4***	***60.9***
	ppv	***42.4***	37.4	***40.9***	***52.4***	***42.8***
	npv	*71.2*	76.8	*72.0*	***84.1***	*75.3*
	auc	55.3	*62.8*	57.1	70.5	61.4
	bar	53.8	***58.5***	54.7	65.9	58.2
	sen	***56.3***	72.0	*48.1*	71.1	***61.9***
up	spc	51.3	***45.1***	61.3	60.7	54.6
	acc	52.9	***53.9***	57.0	64.1	57.0
	ppv	36.1	***39.0***	37.9	47.0	39.9
	npv	70.7	*76.9*	70.9	81.3	74.7

$B|La|H.Lb$

Table S12. Typed (tp) vs. untyped (un) predictor performance.

		lumA	lumB	basal	Her2	overall
	auc	*56.8*	***65.0***	***60.6***	*72.2*	***63.6***
	bar	*54.8*	***60.8***	***56.7***	***68.7***	***60.2***
	sen	55.6	***71.7***	44.6	***74.8***	61.7
tp	spc	***53.9***	*49.8*	***68.8***	***62.7***	***58.8***
	acc	***54.5***	***57.0***	***60.9***	***66.6***	***59.7***
	ppv	*37.1*	***41.2***	***40.9***	***49.5***	***42.2***
	npv	71.4	***78.4***	*72.0*	***83.6***	***75.9***
	auc	56.4	60.6	57.1	71.2	61.3
	bar	54.8	57.0	54.7	66.6	58.3
	sen	***60.1***	66.4	*48.1*	72.2	*61.7*
up	spc	49.4	47.5	61.3	61.0	54.8
	acc	52.9	53.7	57.0	64.7	57.1
	ppv	36.8	38.1	37.9	47.6	39.9
	npv	*71.8*	74.7	70.9	81.9	74.7

$B|Lb|H.La$

Table S13. Typed (tp) vs. untyped (un) predictor performance.

		lumA	lumB	basal	Her2	overall
	auc	***61.5***	*64.0*	***61.6***	***74.7***	***66.1***
	bar	***56.3***	*59.4*	56.3	***71.5***	***60.9***
	sen	37.5	67.7	43.3	***75.9***	56.1
tp	spc	***75.1***	***51.2***	***69.4***	***67.2***	***65.7***
	acc	***62.8***	***56.6***	*60.8*	***70.0***	***62.6***
	ppv	***42.4***	***40.5***	*40.9*	***52.9***	***44.3***
	npv	*71.2*	76.8	71.6	***85.1***	***75.5***
	auc	55.3	63.2	59.5	65.9	60.5
	bar	53.8	59.0	*56.9*	61.7	57.9
	sen	***56.3***	***72.7***	***47.8***	67.0	***61.0***
up	spc	51.3	45.3	66.0	56.5	54.8
	acc	52.9	54.3	60.1	59.9	56.8
	ppv	36.1	39.4	40.8	43.1	39.6
	npv	70.7	*77.6*	*72.3*	77.9	74.3

$H|La|B.Lb$

Table S14. Typed (tp) vs. untyped (un) predictor performance.

		lumA	lumB	basal	Her2	overall
	auc	55.7	***65.0***	57.0	***74.7***	***64.3***
	bar	54.9	***60.8***	54.0	***71.5***	***60.3***
	sen	*63.9*	***71.7***	42.6	***75.9***	***63.5***
tp	spc	45.8	*49.8*	65.3	***67.2***	***57.0***
	acc	51.7	***57.0***	57.9	***70.0***	***59.1***
	ppv	36.7	***41.2***	37.3	***52.9***	***41.8***
	npv	72.2	***78.4***	70.2	***85.1***	***76.3***
	auc	*56.7*	60.6	***59.8***	65.9	60.3
	bar	*55.3*	57.0	***56.2***	61.7	57.6
	sen	61.4	66.4	***46.7***	67.0	60.4
up	spc	***49.2***	47.5	*65.6*	56.5	54.7
	acc	***53.2***	53.7	***59.4***	59.9	56.6
	ppv	*37.2*	38.1	***40.0***	43.1	39.4
	npv	*72.5*	74.7	***71.8***	77.9	74.0

$H|Lb|B.La$

Table S15. Typed (tp) vs. untyped (un) predictor performance.

		lumA	lumB	basal	Her2	overall
	auc	***61.5***	***65.0***	*60.5*	***74.4***	***64.8***
	bar	***56.3***	***60.8***	*57.1*	***69.9***	***61.0***
	sen	37.5	***71.7***	44.3	*72.6*	56.5
tp	spc	***75.1***	*49.8*	***69.8***	***67.1***	***65.4***
	acc	***62.8***	***57.0***	***61.5***	***68.9***	***62.5***
	ppv	***42.4***	***41.2***	*41.7*	***51.8***	***44.3***
	npv	*71.2*	***78.4***	72.0	***83.4***	***75.6***
	auc	55.3	60.6	59.8	71.4	61.8
	bar	53.8	57.0	56.8	66.4	58.5
	sen	***56.3***	66.4	***47.7***	72.2	***60.7***
up	spc	51.3	47.5	65.9	60.6	56.3
	acc	52.9	53.7	59.9	64.4	57.7
	ppv	36.1	38.1	40.6	47.3	40.4
	npv	70.7	74.7	*72.2*	81.8	74.6

$La|Lb|B.H$

Table S16. Typed (tp) vs. untyped (un) predictor performance.

		lumA	lumB	basal	Her2	overall
	auc	***61.5***	***65.0***	***60.6***	***74.7***	***66.1***
	bar	***56.3***	***60.8***	***56.7***	***71.5***	***61.3***
	sen	37.5	***71.7***	44.6	***75.9***	57.4
tp	spc	***75.1***	*49.8*	***68.8***	***67.2***	***65.2***
	acc	***62.8***	***57.0***	***60.9***	***70.0***	***62.7***
	ppv	***42.4***	***41.2***	***40.9***	***52.9***	***44.5***
	npv	*71.2*	***78.4***	*72.0*	***85.1***	***75.9***
	auc	55.3	60.6	57.1	65.9	59.4
	bar	53.8	57.0	54.7	61.7	56.8
	sen	***56.3***	66.4	*48.1*	67.0	***59.5***
up	spc	51.3	47.5	61.3	56.5	54.1
	acc	52.9	53.7	57.0	59.9	55.9
	ppv	36.1	38.1	37.9	43.1	38.7
	npv	70.7	74.7	70.9	77.9	73.3

$B|H|La|Lb$

Performance tables Nearest Centroid on unbalanced compendium

Table S17. Typed (tp) vs. untyped (un) predictor performance.

		lumA	lumB	basal	Her2	overall
	auc	68.6	72.7	50.4	60.6	69.6
	bar	51.8	63.2	49.5	58.1	65.1
	sen	5.8	87.8	86.8	84.9	72.0
tp	spc	97.9	38.6	12.2	31.3	58.2
	acc	85.6	53.5	39.3	48.8	61.8
	ppv	29.9	38.4	36.1	37.5	37.3
	npv	87.1	87.9	62.1	80.9	85.8
	auc	68.6	72.7	50.4	60.6	69.6
	bar	51.8	63.2	49.5	58.1	65.1
	sen	5.8	87.8	86.8	84.9	72.0
up	spc	97.9	38.6	12.2	31.3	58.2
	acc	85.6	53.5	39.3	48.8	61.8
	ppv	29.9	38.4	36.1	37.5	37.3
	npv	87.1	87.9	62.1	80.9	85.8

B.H.La.Lb

Table S18. Typed (tp) vs. untyped (un) predictor performance.

		lumA	lumB	basal	Her2	overall
	auc	*68.4*	72.1	***62.2***	59.3	69.7
	bar	*52.2*	61.2	***58.0***	57.0	64.0
	sen	6.7	***90.9***	50.0	*83.9*	64.2
tp	spc	***97.8***	31.5	***66.1***	30.0	***63.8***
	acc	***85.6***	49.5	***60.2***	47.7	***63.9***
	ppv	***32.9***	36.6	***45.6***	36.9	***38.0***
	npv	*87.2*	***88.9***	***69.9***	79.3	83.8
	auc	68.0	*72.2*	50.4	***61.3***	***70.0***
	bar	52.0	***62.7***	50.9	***58.0***	***64.7***
	sen	*6.9*	86.1	***81.7***	83.2	***70.0***
up	spc	97.0	***39.2***	20.1	***32.8***	59.4
	acc	85.0	***53.4***	42.4	***49.3***	62.1
	ppv	27.3	***38.1***	36.8	***37.6***	37.3
	npv	87.1	86.7	65.6	*80.0*	***85.2***

B|H.La.Lb

Table S19. Typed (tp) vs. untyped (un) predictor performance.

		lumA	lumB	basal	Her2	overall
	auc	*68.6*	71.9	46.4	***74.7***	***70.1***
	bar	*52.1*	***64.5***	48.1	***71.5***	***66.8***
	sen	5.5	***87.7***	*86.7*	*75.9*	***70.5***
tp	spc	***98.6***	***41.3***	9.6	***67.2***	***63.0***
	acc	***86.2***	***55.4***	37.6	***70.0***	***64.9***
	ppv	***40.2***	***39.4***	35.3	***52.9***	***39.7***
	npv	*87.2*	***88.6***	55.8	***85.1***	***86.1***
	auc	68.3	***72.4***	***50.3***	60.3	69.4
	bar	52.0	62.8	***49.6***	57.5	64.6
	sen	***6.5***	86.5	86.5	74.9	70.0
up	spc	97.5	39.1	***12.6***	40.2	59.3
	acc	85.4	53.5	***39.5***	51.5	62.0
	ppv	29.2	38.2	***36.1***	37.9	37.3
	npv	87.1	86.9	***62.3***	76.7	85.1

$H|B.La.Lb$

Table S20. Typed (tp) vs. untyped (un) predictor performance.

		lumA	lumB	basal	Her2	overall
	auc	***64.8***	65.6	***63.4***	***76.5***	67.3
	bar	***56.3***	59.3	***58.3***	***71.2***	62.9
	sen	***31.3***	74.1	49.6	74.1	60.2
tp	spc	81.3	***44.5***	***67.1***	***68.4***	***65.7***
	acc	74.6	53.5	***60.7***	***70.3***	***64.3***
	ppv	20.5	36.8	***46.2***	***53.3***	***37.7***
	npv	***88.5***	79.8	***70.0***	***84.4***	82.7
	auc	63.0	***72.1***	49.7	61.2	***68.8***
	bar	54.6	***62.7***	49.6	58.4	***64.3***
	sen	19.9	***85.8***	***85.6***	***83.0***	***73.2***
up	spc	***89.2***	39.6	13.6	33.8	55.5
	acc	***80.0***	*53.6*	39.8	49.9	60.0
	ppv	***22.4***	***38.2***	36.1	37.9	36.2
	npv	87.9	***86.5***	62.5	80.4	***85.7***

$La|B.H.Lb$

Table S21. Typed (tp) vs. untyped (un) predictor performance.

		lumA	lumB	basal	Her2	overall
	auc	65.9	***71.9***	46.7	56.3	67.5
	bar	51.5	***64.7***	48.6	57.2	***64.9***
	sen	4.8	74.6	***89.5***	***84.2***	67.0
tp	spc	***98.2***	***54.7***	7.8	30.3	***62.8***
	acc	***85.7***	***60.7***	37.4	47.9	***63.9***
	ppv	***28.7***	***41.8***	35.6	37.0	***38.4***
	npv	87.0	83.2	56.2	79.8	84.6
	auc	***67.1***	70.2	***50.1***	***61.2***	***68.9***
	bar	***52.3***	62.3	***49.8***	***58.2***	64.4
	sen	***8.6***	***82.7***	85.6	82.3	***69.8***
up	spc	96.0	41.9	***14.0***	***34.0***	59.1
	acc	84.3	54.3	***40.0***	***49.8***	61.8
	ppv	25.1	38.3	***36.2***	***37.8***	37.1
	npv	***87.2***	***84.8***	***62.7***	*79.9*	***85.0***

$Lb|B.H.La$

Table S22. Typed (tp) vs. untyped (un) predictor performance.

		lumA	lumB	basal	Her2	overall
	auc	***69.0***	***72.6***	***61.7***	***74.9***	***71.1***
	bar	*52.5*	***63.1***	***57.7***	***70.4***	***65.9***
	sen	6.4	***92.4***	47.4	71.8	62.2
tp	spc	***98.6***	33.7	***68.0***	***69.0***	***69.5***
	acc	***86.3***	51.5	***60.6***	***69.9***	***67.6***
	ppv	***42.8***	37.8	***45.8***	***53.0***	***41.4***
	npv	*87.3*	***91.1***	***69.4***	***83.4***	84.2
	auc	67.5	71.6	49.9	61.3	69.6
	bar	52.2	62.4	50.3	58.2	64.5
	sen	***8.3***	84.8	***83.6***	***80.3***	***69.8***
up	spc	96.1	***40.0***	16.9	36.0	59.2
	acc	84.4	***53.6***	41.1	50.5	61.9
	ppv	25.6	***38.1***	36.5	38.0	37.1
	npv	87.2	85.8	64.2	78.9	***85.0***

$B.H|La.Lb$

Table S23. Typed (tp) vs. untyped (un) predictor performance.

		lumA	lumB	basal	Her2	overall
	auc	64.9	65.2	45.6	***73.6***	68.1
	bar	*53.0*	59.3	48.8	***70.3***	***65.0***
	sen	8.3	75.8	***95.4***	74.9	68.1
tp	spc	***97.7***	***42.9***	2.1	***65.7***	***61.9***
	acc	***85.8***	52.8	36.0	***68.7***	***63.5***
	ppv	***35.9***	36.6	35.7	***51.5***	***38.2***
	npv	*87.4*	80.3	45.1	***84.3***	84.9
	auc	***66.2***	***71.2***	***50.1***	62.0	***69.3***
	bar	52.7	***62.7***	***49.8***	59.1	64.6
	sen	***11.0***	***84.5***	84.9	***82.6***	***70.8***
up	spc	94.5	40.8	***14.7***	35.6	58.4
	acc	83.3	***54.1***	***40.2***	51.0	61.6
	ppv	23.9	***38.4***	***36.2***	38.5	37.0
	npv	87.3	***85.9***	***62.8***	80.9	***85.3***

$B.La|H.Lb$

Table S24. Typed (tp) vs. untyped (un) predictor performance.

		lumA	lumB	basal	Her2	overall
	auc	***68.4***	68.0	***64.8***	59.2	***70.2***
	bar	***54.5***	***63.4***	***60.8***	54.3	***64.8***
	sen	*13.8*	66.8	63.6	***94.7***	60.7
tp	spc	***95.3***	***59.9***	***57.9***	13.8	***69.0***
	acc	***84.4***	***62.0***	***60.0***	40.3	***66.9***
	ppv	***31.1***	***42.1***	***46.3***	34.8	***40.4***
	npv	***87.8***	80.6	***73.7***	***84.6***	83.6
	auc	65.4	***71.6***	49.9	***61.7***	69.1
	bar	53.1	62.6	49.8	***58.3***	64.4
	sen	13.1	***84.8***	***85.1***	80.5	***71.1***
up	spc	93.2	40.4	14.4	***36.1***	57.7
	acc	82.5	53.9	40.1	***50.6***	61.2
	ppv	23.0	38.3	36.2	***38.0***	36.7
	npv	87.5	***86.0***	62.8	79.2	***85.2***

$B.Lb|H.La$

Table S25. Typed (tp) vs. untyped (un) predictor performance.

		lumA	lumB	basal	Her2	overall
tp	auc	***69.0***	***72.6***	***62.2***	***74.7***	***71.8***
	bar	*52.5*	***63.1***	***58.0***	***71.5***	***66.3***
	sen	6.4	***92.4***	50.0	*75.9*	63.5
	spc	***98.6***	33.7	***66.1***	***67.2***	***69.0***
	acc	***86.3***	51.5	***60.2***	***70.0***	***67.6***
	ppv	***42.8***	37.8	***45.6***	***52.9***	***41.5***
	npv	*87.3*	***91.1***	***69.9***	***85.1***	84.5
up	auc	67.5	71.6	50.4	60.3	69.6
	bar	52.2	62.4	50.9	57.5	64.3
	sen	***8.3***	84.8	***81.7***	74.9	***68.4***
	spc	96.1	***40.0***	20.1	40.2	60.1
	acc	84.4	***53.6***	42.4	51.5	62.3
	ppv	25.6	***38.1***	36.8	37.9	37.2
	npv	87.2	85.8	65.6	76.7	*84.7*

$B|H|La.Lb$

Table S26. Typed (tp) vs. untyped (un) predictor performance.

		lumA	lumB	basal	Her2	overall
tp	auc	***64.8***	65.2	***62.2***	***73.6***	68.0
	bar	***56.3***	59.3	***58.0***	***70.3***	62.9
	sen	***31.3***	75.8	50.0	74.9	61.1
	spc	81.3	***42.9***	***66.1***	***65.7***	***64.7***
	acc	74.6	52.8	***60.2***	***68.7***	***63.8***
	ppv	20.5	36.6	***45.6***	***51.5***	***37.4***
	npv	***88.5***	80.3	***69.9***	***84.3***	82.8
up	auc	63.0	***71.2***	50.4	62.0	***69.0***
	bar	54.6	***62.7***	50.9	59.1	***64.4***
	sen	19.9	***84.5***	***81.7***	***82.6***	***71.7***
	spc	***89.2***	40.8	20.1	35.6	57.1
	acc	***80.0***	***54.1***	42.4	51.0	60.8
	ppv	***22.4***	***38.4***	36.8	38.5	36.6
	npv	87.9	***85.9***	65.6	80.9	***85.4***

$B|La|H.Lb$

Table S27. Typed (tp) vs. untyped (un) predictor performance.

		lumA	lumB	basal	Her2	overall
	auc	***68.4***	***71.9***	***62.2***	59.2	***69.4***
	bar	***54.5***	***64.7***	***58.0***	54.3	*64.5*
	sen	*13.8*	74.6	50.0	***94.7***	60.5
tp	spc	***95.3***	***54.7***	***66.1***	13.8	***68.5***
	acc	***84.4***	***60.7***	***60.2***	40.3	***66.5***
	ppv	***31.1***	***41.8***	***45.6***	34.8	***39.9***
	npv	***87.8***	83.2	***69.9***	***84.6***	83.4
	auc	65.4	70.2	50.4	***61.7***	69.0
	bar	53.1	62.3	50.9	***58.3***	64.2
	sen	13.1	***82.7***	***81.7***	80.5	***69.4***
up	spc	93.2	41.9	20.1	***36.1***	59.1
	acc	82.5	54.3	42.4	***50.6***	61.7
	ppv	23.0	38.3	36.8	***38.0***	36.9
	npv	87.5	***84.8***	65.6	79.2	***84.8***

$B|Lb|H.La$

Table S28. Typed (tp) vs. untyped (un) predictor performance.

		lumA	lumB	basal	Her2	overall
	auc	***64.8***	68.0	***64.8***	***74.7***	***70.4***
	bar	***56.3***	***63.4***	***60.8***	***71.5***	***65.1***
	sen	***31.3***	66.8	63.6	*75.9*	60.9
tp	spc	81.3	***59.9***	***57.9***	***67.2***	***69.2***
	acc	74.6	***62.0***	***60.0***	***70.0***	***67.1***
	ppv	20.5	***42.1***	***46.3***	***52.9***	***40.6***
	npv	***88.5***	80.6	***73.7***	***85.1***	83.7
	auc	63.0	***71.6***	49.9	60.3	68.4
	bar	54.6	62.6	49.8	57.5	64.0
	sen	19.9	***84.8***	***85.1***	74.9	***71.4***
up	spc	***89.2***	40.4	14.4	40.2	56.6
	acc	***80.0***	53.9	40.1	51.5	60.4
	ppv	***22.4***	38.3	36.2	37.9	36.2
	npv	87.9	***86.0***	62.8	76.7	***85.1***

$H|La|B.Lb$

Table S29. Typed (tp) vs. untyped (un) predictor performance.

		lumA	lumB	basal	Her2	overall
	auc	64.9	***71.9***	45.6	***74.7***	***69.7***
	bar	*53.0*	***64.7***	48.8	***71.5***	***66.9***
	sen	8.3	74.6	***95.4***	*75.9*	67.8
tp	spc	***97.7***	***54.7***	2.1	***67.2***	***65.9***
	acc	***85.8***	***60.7***	36.0	***70.0***	***66.4***
	ppv	***35.9***	***41.8***	35.7	***52.9***	***40.7***
	npv	*87.4*	83.2	45.1	***85.1***	***85.6***
	auc	***66.2***	70.2	***50.1***	60.3	68.7
	bar	52.7	62.3	***49.8***	57.5	64.1
	sen	***11.0***	***82.7***	84.9	74.9	***68.9***
up	spc	94.5	41.9	***14.7***	40.2	59.2
	acc	83.3	54.3	***40.2***	51.5	61.7
	ppv	23.9	38.3	***36.2***	37.9	36.9
	npv	87.3	***84.8***	***62.8***	76.7	84.6

$H|Lb|B.La$

Table S30. Typed (tp) vs. untyped (un) predictor performance.

		lumA	lumB	basal	Her2	overall
	auc	***64.8***	***71.9***	***61.7***	***74.9***	68.2
	bar	***56.3***	***64.7***	***57.7***	***70.4***	***64.4***
	sen	***31.3***	74.6	47.4	71.8	59.5
tp	spc	81.3	***54.7***	***68.0***	***69.0***	***69.2***
	acc	74.6	***60.7***	***60.6***	***69.9***	***66.7***
	ppv	20.5	***41.8***	***45.8***	***53.0***	***40.1***
	npv	***88.5***	83.2	***69.4***	***83.4***	83.2
	auc	63.0	70.2	49.9	61.3	*68.4*
	bar	54.6	62.3	50.3	58.2	64.0
	sen	19.9	***82.7***	***83.6***	***80.3***	***71.0***
up	spc	***89.2***	41.9	16.9	36.0	57.0
	acc	***80.0***	54.3	41.1	50.5	60.6
	ppv	***22.4***	38.3	36.5	38.0	36.3
	npv	87.9	***84.8***	64.2	78.9	***85.1***

$La|Lb|B.H$

Table S31. Typed (tp) vs. untyped (un) predictor performance.

		lumA	lumB	basal	Her2	overall
tp	auc	***64.8***	***71.9***	***62.2***	***74.7***	***69.9***
	bar	***56.3***	***64.7***	***58.0***	***71.5***	***64.8***
	sen	***31.3***	74.6	50.0	*75.9*	60.8
	spc	81.3	***54.7***	***66.1***	***67.2***	***68.8***
	acc	74.6	***60.7***	***60.2***	***70.0***	***66.7***
	ppv	20.5	***41.8***	***45.6***	***52.9***	***40.2***
	npv	***88.5***	83.2	***69.9***	***85.1***	83.5
up	auc	63.0	70.2	50.4	60.3	68.3
	bar	54.6	62.3	50.9	57.5	63.8
	sen	19.9	***82.7***	***81.7***	74.9	***69.7***
	spc	***89.2***	41.9	20.1	40.2	57.9
	acc	***80.0***	54.3	42.4	51.5	60.9
	ppv	***22.4***	38.3	36.8	37.9	36.4
	npv	87.9	***84.8***	65.6	76.7	***84.7***

$B|H|La|Lb$
